# Light and Dark Cycles Control the Structural Evolution of Photoresponsive Supramolecular Systems

**DOI:** 10.1002/anie.4843934

**Published:** 2026-06-02

**Authors:** Alejandro Méndez‐Ardoy, Nicolas Cissé, Adrian Sanchez‐Fernandez, Patricia Fulias‐Guzmán, Marc C. A. Stuart, Tibor Kudernac, Javier Montenegro

**Affiliations:** ^1^ Departamento de Química Orgánica Facultad de Química Universidad de Sevilla Sevilla Spain; ^2^ Instituto de Investigaciones Químicas (IIQ) CSIC–Universidad de Sevilla Sevilla Spain; ^3^ Stratingh Institute for Chemistry University of Groningen Groningen the Netherlands; ^4^ Centro Singular de Investigación en Química Biolóxica e Materiais Moleculares (CIQUS) Departamento de Enxeñaría Química, Universidade de Santiago de Compostela Santiago de Compostela Spain; ^5^ Centro Singular de Investigación en Química Biolóxica e Materiais Moleculares (CIQUS) Departamento de Química Orgánica Universidade de Santiago de Compostela Santiago de Compostela Spain

**Keywords:** photoswitches, self‐assembly, supramolecular chemistry

## Abstract

As the supply of light on Earth is cyclic and asymmetric, living systems that rely on photochemical energy have adapted to accommodate periods of light and darkness. During the day, light energy is available, while throughout the dark phases, thermal relaxation processes can be used to increase functional and structural organization. While light‐driven supramolecular assembly has been reported, structural adaptation under alternating light/dark input remains largely unexplored in synthetic supramolecular systems. Here, we show how an oscillating light energy supply can facilitate the structural selection of self‐assembling photoswitchable peptides compared to continuous illumination. We demonstrate that polymorphic self‐assembled structures are transiently formed and sustained only under light irradiation, while alternating periods of irradiation and darkness favor the formation of a thermodynamically more stable supramolecular architecture. These findings demonstrate the key role that rest (darkness) periods can have in the self‐assembly pathway selection of molecular and self‐organized supramolecular photosystems.

## Graphical abstract

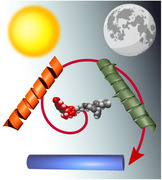

## 1 Introduction

Living systems have adapted to a world of alternating light and darkness, adjusting to the changing patterns of daily sunlight [1, 2]. Indeed, this asymmetric and alternating input of light energy should have played a key functional role in early photoresponsive molecules. Such environmental pressure led to the emergence of mechanisms in which a phase of accumulation of energy is followed by a dark (resting) phase where this energy can be transformed. It is therefore not unusual to find systems that are able to anticipate and respond to these oscillations between the light and dark phases [3]. In photochemical processes, the dark phases provide an optimal scenario for transforming light energy and structural intermediates [4]. For example, this is observed in the thermal relaxation of high‐energy protein complexes formed after photon absorption [5]. Likewise, throughout the dark phases, photosensitive molecules undergo configurational isomerization that rearranges their intra‐ and intermolecular interactions. Thus, it could be hypothesized that an alternation of light energy inputs would enable the selection of different structural assembly pathways in the potential energy landscape, with different kinetic intermediates, which can be typically observed in self‐assembling supramolecular networks [6, 7, 8, 9, 10, 11, 12, 13, 14, 15]. More specifically, for molecular systems undergoing light‐induced supramolecular transformations [16, 17, 18], the sequential disruption of the light energy supply should enable, or even select, certain self‐organizing pathways. Nevertheless, these types of systems’ mechanisms of structural adaptation and self‐selection under alternating energy supply have remained elusive for synthetic supramolecular systems [19]. Here, we report a photoswitchable synthetic peptide that, under light energy cycles, exhibits supramolecular polymorphism coupled to darkness‐driven structural self‐selection. We demonstrate that light sustains a transient assembled state in the form of helical nanoribbons, hindering the system from undergoing a transition to the disassembled state, which is observed after long periods of darkness. However, one critical finding is that during short periods of dark phases, the light‐adapted state relaxes into previously inaccessible supramolecular intermediate architectures, collapsing nanoribbons that facilitate a structural transition from the pool of helical nanoshapes to a uniform nanotubular architecture.

**FIGURE 1:**
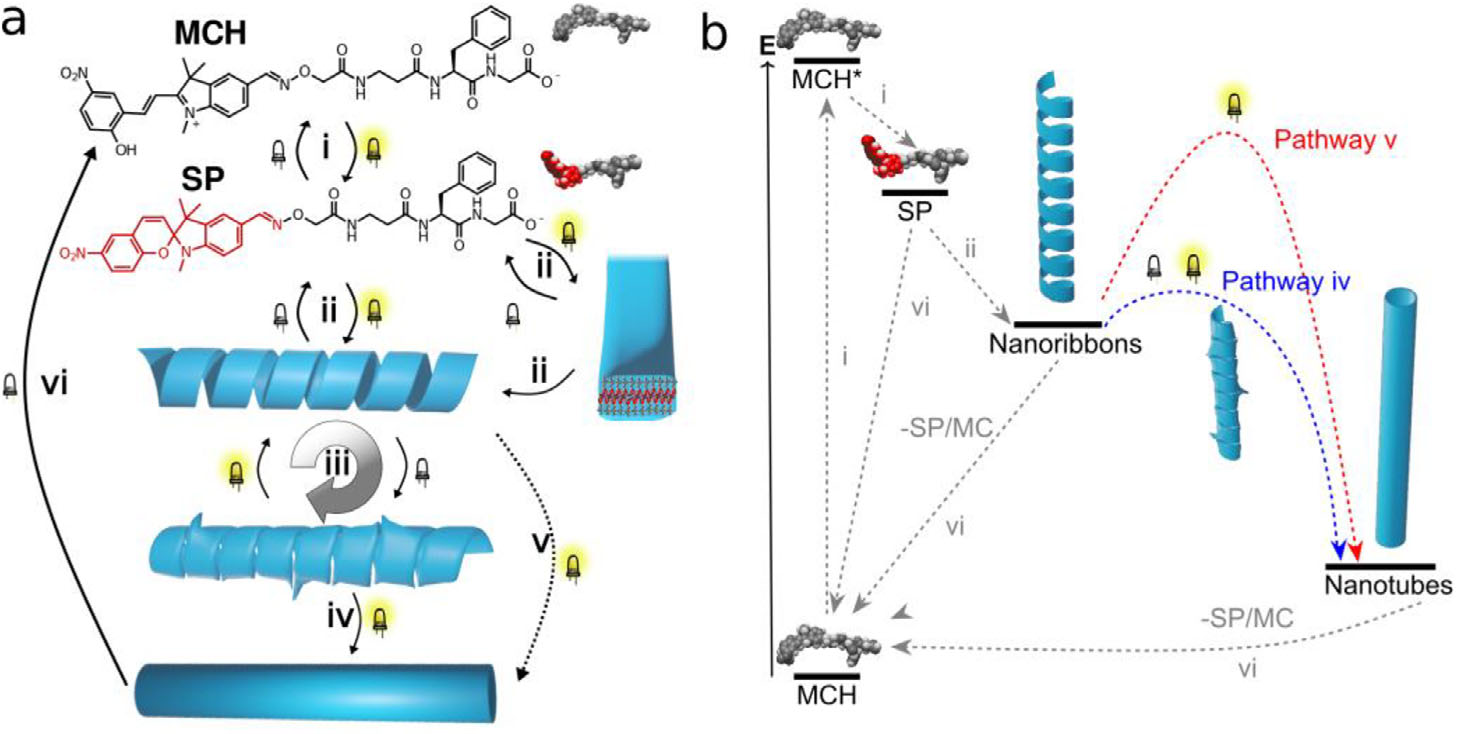
Structural selection of supramolecular polymorphs under light energy input oscillations (a) self‐assembly modulation by light and (b) energy landscape for the cyclic photoswitch assembly. (i) Light‐triggered cycle of isomerization and thermal relaxation. (ii) self‐assembly of the light‐adapted state spiropyran (SP) leads to initial short crescent nanotapes that, under continuous irradiation, evolved into elongated nanoribbons. (iii) Stopping the light energy flow causes the collapse of the nanoribbons by the disassembly of the merocyanine formed upon thermal relaxation (**MCH**). (iv) Light‐dark cycles favor the transformation of the helical nanoribbon populations into a final nanotubular architecture. (v) A higher activation energy hinders the nanoribbons to evolve to the nanotubular architecture. (vi) In the absence of light, the nanotubes dissolve into the water‐soluble **MCH** formed by thermal relaxation, confirming their transient nature.

The here‐introduced spiropyran‐based [20, 21] photoswitchable peptide isomerizes from a soluble monomeric state enriched in the charged merocyanine form in the dark to a more hydrophobic spiropyran state under visible light (Figure 1). Light irradiation triggered a molecular reconfiguration that resulted in the gradual self‐assembly of the peptide‐spiropyran monomer into chiral helical nanoribbons showing transient assembly dynamics [22]. In the absence of the light supply, the re‐isomerization of the spiropyran to the more soluble merocyanine monomers prompted the loss of helicity and the disassembly and disappearance of the supramolecular crescent tapes. However, an additional input of light energy recovers the originally formed helical tapes. In the dark, thermal re‐isomerization caused the relaxation of the supramolecular assemblies and the catastrophic collapse of the helical nanoribbons into distorted helices. Sustained cycling between light and dark periods drove the system toward increased formation of the nanotubular structure, which is lower in energy yet remains transient. The statistically confirmed increased production of the lower energy nanotubular polymorph, under alternating dark periods, demonstrates that oscillations in the light supply can lead to divergent self‐assembly pathways and to spontaneous structural self‐selection in supramolecular systems.

## 2 Results and Discussion

### 2.1 Light‐Triggered Isomerization

The design for the light‐triggered supramolecular system consists of a short peptide monomer that is covalently connected to a merocyanine/spiropyran photoswitch (Figure 1). This particular sequence of three amino acids (*β*Ala‐Phe‐Gly) was selected because it provided well‐defined assemblies in comparison to other sequence mutations (Figures S3–S5). For example, we found that the substitution of the hydrophobic amino acid, phenylalanine for valine or alanine, could lead to poorly defined assemblies. On the other hand, we also confirmed that the order of amino acids in the *β*‐sheet region (e.g., Phe and Gly) was key to secure optimal amphiphilic character and achieve regular self‐assembly (Figure S3). The presence of Phe alone is not sufficient to obtain ordered supramolecular structures; its position within the short peptide sequence is also critical. In *β*Ala‐Phe‐Gly, the Phe residue is positioned closer to the spiropyran unit, which likely promotes an organized hydrophobic/aromatic domain within the bilayer‐like assembly while leaving the more polar peptide terminus exposed to water. Swapping Gly and Phe to give *β*Ala‐Gly‐Phe disrupts this spatial arrangement and results in poorly defined aggregates rather than high‐order nanostructures. Consistently, the CD spectrum of *β*Ala‐Gly‐Phe shows only a weak and markedly different Cotton effect compared with the pronounced supramolecular CD response of *β*Ala‐Phe‐Gly, indicating that the spiropyran chromophores are not organized into the same chiral exciton‐coupled packing arrangement. These results show that ordered assembly depends not only on the hydrophobic/aromatic contribution of Phe, but also on its correct presentation within the amphiphilic peptide sequence. Irradiation experiments in the peptide mutants (Figure S4) clearly show that the photoswitching is general in the series, whereas a well‐defined supramolecular response is sequence specific. This behavior is likely due to the higher hydrophobicity and *π*–*π* interactions caused by the presence of the Phe residue for **SP** [23].

**FIGURE 2:**
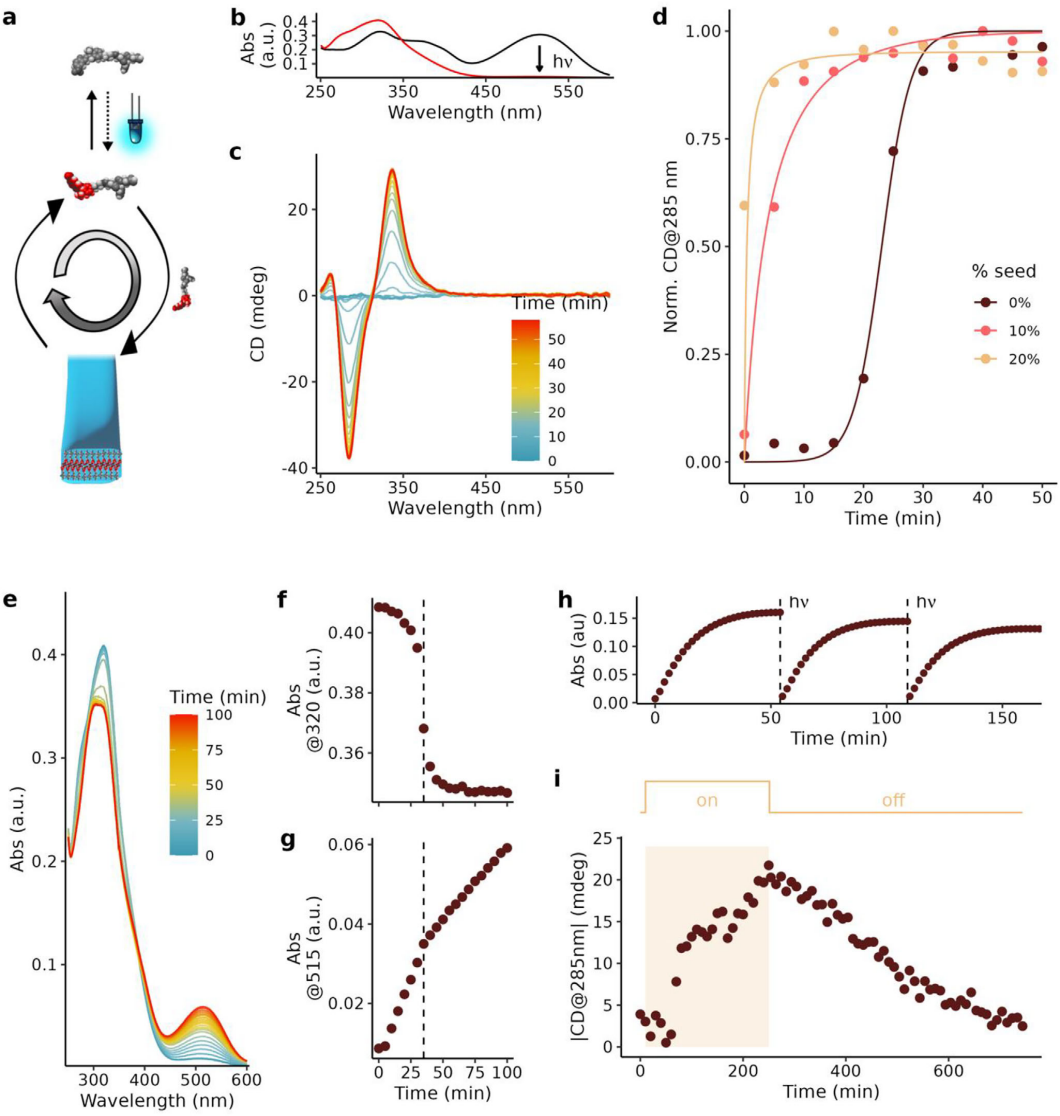
Spectroscopic characterization of the photoswitchable peptide under light irradiation: (a) Assembly mechanism and bilayer supramolecular packing of the peptide photoswitch assembly (MES 20 mM, pH 6, path length: 0.2 cm, 20°C). (b) UV–vis spectrum acquired from **MCH/SP** solution (110 µM) before (black) and after irradiation with visible light (red). (c) Exciton spectra monitored in time after the conversion of initial **MCH** to **SP** by visible light irradiation (180 µM) after a single light pulse (10–15 s irradiation with broadband white light, 38 cd). (d) Self‐assembly monitored in the presence of preassembled seeds (total peptide concentration 110 µM). (e) Evolution of UV/vis absorption spectra after **SP** conversion (110 µM). (f and g) show the profiles of the evolution of **SP** + **MCH** (320 nm) and **MCH** (515 nm), respectively. (h) Transient response of the peptide photoswitch at 45°C: cycled thermal isomerization (at 110 µM) monitored by UV/vis spectroscopy (515 nm band). The dashed lines indicate the time of the irradiation pulses (broadband white light, 10–15 s, 38 cd, path length is 0.2 cm). (i) Sustained self‐assembly under continuous broadband white light irradiation (110 µM, 30°C, 10 cd).

Light irradiation affects these intermolecular forces, as the photoswitch isomerizes from the water‐soluble protonated merocyanine (**MCH**) to a more hydrophobic spiropyran form (**SP**) [24]. Light pulses can be used to control the rate of the ring‐closing isomerization, effectively bypassing the microscopic reversibility principle [25, 26, 27, 28] and allowing precise control of the **SP**/**MCH** populations [29, 30, 31, 32, 33, 34, 35, 36, 37, 38]. To prevent any assembly changes associated with pH variations, such as glycine protonation triggered by the photoacidity of the spiropyran, experiments were carried out in MES buffer pH 6.0 at a 20 mM concentration. An equilibrated diluted solution of this monomer in MES buffer (20 mM, pH 6) consists of 27 ± 2% of **MCH** form in the absence of light, as observed by HPLC (Figure S6). Short light pulses were used to probe the transient assembly dynamics, analogous to the addition of a finite amount of chemical fuel to other reported transient systems [22]. After a short light energy pulse (ca. 15 s, 38 cd), a photostationary state was reached, where the ultraviolet‐visible absorption spectra show the complete depletion of the band at 515 nm attributed to the **MCH** form and the increase of the **SP** band, indicating full conversion (Figures 2b and S7). ^1^H NMR experiments revealed quantitative conversion of **MCH** into **SP** after light irradiation. In addition, the absence of spiropyran signals at higher fields is consistent with a slower exchange kinetics for the **SP** isomer (Figure S8). Here, we target the states determined by the photostationary state of the **MCH**‐to‐**SP** photo‐isomerization, which amounts to the full conversion. Upon light irradiation, the increased concentration of **SP** surpasses the critical self‐assembly concentration, and a supramolecular polymerization starts, as observed from the appearance of the corresponding exciton coupling in the circular dichroism (CD) absorption spectra (Figure 2c). An estimated association constant for the supramolecular polymerization (*K*_a_) of the **SP** was calculated to be in the order of 10^4^ M^−1^ according to the UV–vis concentration dependence (Figure S9a,b). A 30 µM critical aggregation concentration (CAC) could also be estimated by the concentration threshold of the relative **SP** UV–vis bands absorbances versus the peptide concentration (Figure S9c). The profile of the CD spectra after light irradiation showed a concentration‐dependent lag time, which was consistent with a nucleation‐elongation cooperative polymerization mechanism with kinetic constants in the order of 2 × 10^−5^ s^−1^ and 30 M^−1^ s^−1^, respectively (Figure S10a) [39]. This physically autocatalyzed self‐assembly was validated by the gradual shortening of the exciton coupling lag time in the presence of increasing concentrations of previously self‐assembled seeds of spiropyran (Figure 2d).

### 2.2 Thermal Relaxation of the Photo‐Generated Spiropyran State

The next step was to monitor the thermal relaxation of the photo‐generated spiropyran. The reversibility of the process in the absence of light was confirmed by the UV kinetic analysis (Figure 2e). The evolution of the UV intensity at 320 nm, where both forms of the photoswitch absorb significantly, showed a nonlinear sigmoidal profile during the thermal relaxation, which also corroborated the autocatalytic self‐assembly mechanism (Figure 2f). An isosbestic point was not observed since spiropyran undergoes self‐assembly above the critical self‐assembly concentration, resulting in more than two‐species equilibrium (Figure S7). Following the rise of the merocyanine absorption band at 515 nm, a change in the rate of the process can be observed (Figure 2g). The onset regime change is coincident with the sigmoidal transition where the two bands of the photoswitch overlap (Figure 2e,g). After this onset point, the deceleration of the thermal relaxation suggests a stabilization of the spiropyran form in the supramolecular assembly. These observations were supported by the lack of any spectroscopic evidence of the **MCH** co‐assembly—namely, no new excitonic signatures (Figure 2c), no solvatochromism (Figure S11), and no deviations of linearity in the **SP**/**MCH** titrations (Figure S12). Therefore, we can conclude that the **SP** → **MCH** isomerization occurs largely after spiropyran monomer exchange with the aqueous solvent, where the **MCH** formed by thermal relaxation is stabilized through hydrogen bonding with the water [40]. The temperature of the aqueous solution was raised to 45°C to accelerate the thermal relaxation and disassembly. The corresponding CD kinetic traces showed the temperature‐controlled assembly dynamics and also confirmed the presence of transient [41, 42] aggregation states (Figure S13a). Based on the CD and UV–vis data (Figure S13 and Tables S3 and S4), we infer a separation of timescales between molecular isomerization and supramolecular reorganization. In the dark, thermal recovery of the merocyanine form occurs faster than large‐scale restructuring of the assemblies, increasing the fraction of the more soluble isomer and promoting partial removal of molecules and defect formation within the assemblies. Upon renewed irradiation, the regenerated spiropyran is reincorporated into the pre‐existing assemblies more rapidly than new structures can nucleate, consistent with the cooperative assembly process observed. Increasing the thermal isomerization rate to the merocyanine form in the dark accelerates both monomer exchange and switching rate in the aqueous solvent (Figure S13b). Transient assembly dynamics could also be controlled by programmed light irradiation, where we could observe reversible assembly (Figure S14a), stepwise assembly (Figure S14b), and multiple spiropyran‐merocyanine switching cycles (Figure 2h). For a given irradiation wavelength, the photostationary state should remain unchanged once it is reached (typically after a few seconds in our experiments); however, we observed a small degree of photo‐fatigue upon long repeated cycling. The chiral assembly could be sustained by continuous irradiation (Figure 2i), where the photostationary state is composed purely of the **SP** isomer.

### 2.3 Self‐Assembly of the Peptide Photoswitch

**FIGURE 3:**
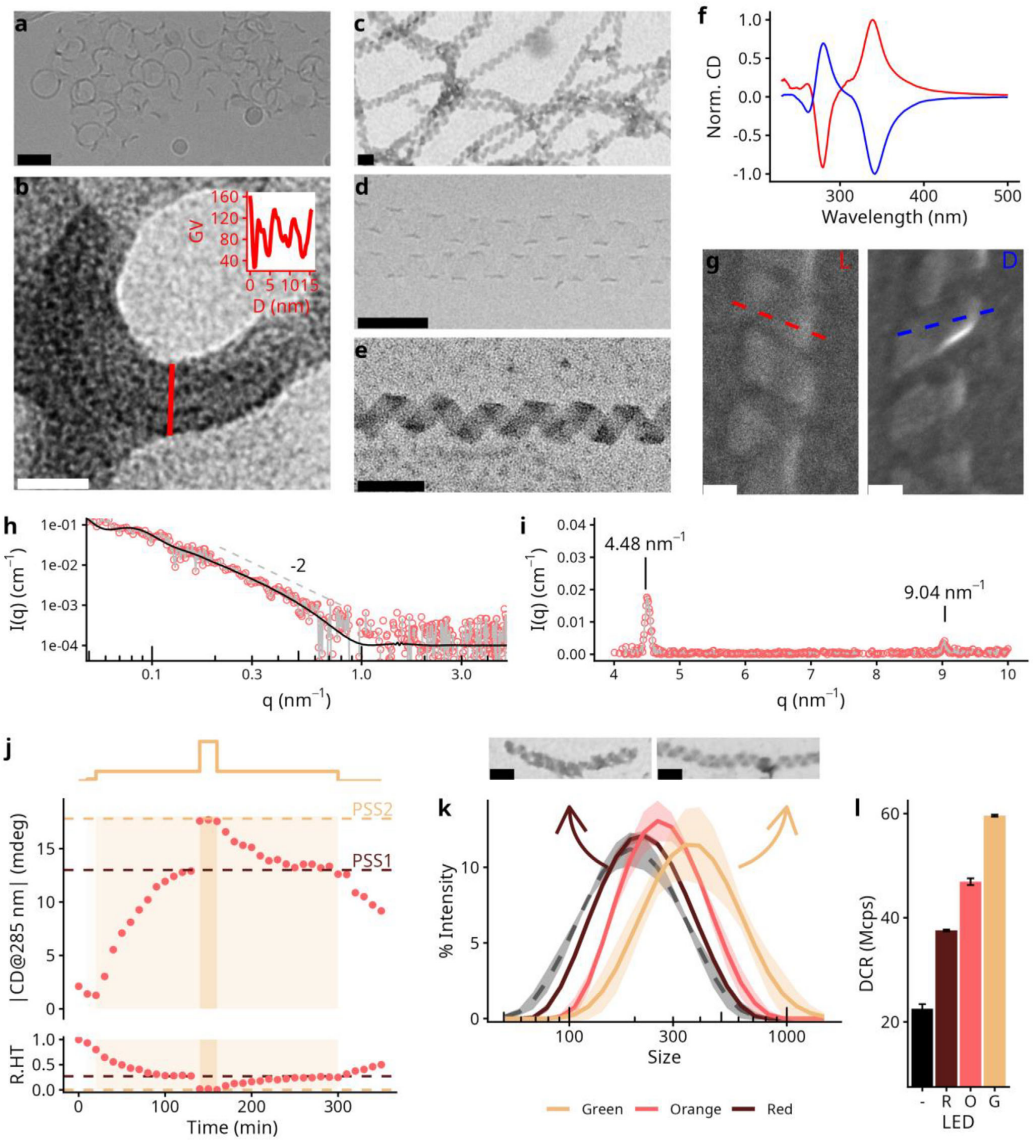
Structural evolution of self‐assembled architecture formed by the spiropyran form under irradiation (0.5 mM, 45°C, MES 50 mM, pH 6). (a) Low magnification cryoTEM image (after 30 min irradiation) showing the initial presence of tape‐shaped assemblies. (b) High‐resolution TEM showing the size of the tape thickness (∼5 nm). (c) STEM micrographs after 3 h of continuous irradiation (broadband white light, 38 cd). (d) CryoTEM and (e) high magnification TEM micrographs showing a magnified area of the helical nanoribbons. (f) Circular dichroism spectra of the d (blue) and l (red) enantiomers of SP after assembly. (g) Helical nanoribbons obtained from the l (left) and d (right) enantiomer. (h) SAXS and (i) WAXS spectra of helical nanoribbons. (j) Real‐time modulation of the helical assembly by tuning the amount of light irradiated (broadband white light, 10 cd) by time unit using short‐spaced light pulses (peptide 125 µM, MES 20 mM pH 6, 30°C). Pulse width modulation of the LED voltage was adjusted to achieve photostationary states (PSS): PSS1 (4% duty cycle) and PSS2 (60% duty cycle); Tuning aggregate size was monitored by DLS by modulation of time length of light pulses (peptide 300 µM, MES 50 mM pH 6, 45 C, 3 h.). (k) DLS data showing the size intensity distribution; insert shows STEM micrographs of samples using two different irradiation conditions at 500 µM concentration. All scale bars correspond to 200 nm; (l) derived DLS count rate (proportional to the size and quantity of nanoassemblies in solution). R, O, and G denote irradiations carried out with commercial red (10 cd, *λ* = 629 nm), orange (7 cd, *λ* = 590 nm), and green (20 cd, *λ* = 525 nm) LEDs, and—denotes no light irradiation.

An increase of the intensity of the light scattered right after the light irradiation pulses was also observed (Figure S10b). The population of immediately formed small aggregates was quantified by dynamic light scattering (DLS) and nanoparticle tracking analysis (NTA) (Figures S15 and S16). These results show an exponential increase of the number of assemblies after the application of the light pulse. Different modes of transmission electron microscopy (STEM, HRTEM, and cryo‐TEM) were next employed to characterize these small assemblies (Figure 3). Before irradiation, cryo‐TEM micrographs of the monomers in aqueous solution showed small dots (< 5 nm), which were compatible with kinetically trapped, low‐order oligomers with similar dimensions to those of a few molecules of the peptide photoswitch (Figure S17a,b). After a pulse of white light (38 cd, 10–15 s), supramolecular structures reminiscent of crescent shapes [43] started clustering in the bulk aqueous media (Figures S17c,d and S18). After a longer irradiation time (e.g., 30 min), a formation of small, curved tapes of defined sections with an average thickness of ∼5 nm could be observed by HRTEM (Figure 3a). Considering the ∼2.3 nm length of the peptide‐spiropyran monomer (Figure S19), the tape thickness was consistent with a bilayer packing of the amphiphilic photoswitch, with the ionized peptide carboxylates exposed to the water environment (Figures 1a and 3b).

### 2.4 Formation of Chiral Polymorphs by One Irradiation Cycle

The nucleation elongation mechanism (Figure 2d–g) suggested that under prolonged irradiation time, the initial small supramolecular structures should grow into higher‐order aggregates. STEM and cryoTEM microscopy analysis of samples irradiated with visible light for 3 h confirmed the evolution of the initial crescent assemblies into much longer helical nanoribbons with lengths beyond 1 µm (Figure 3c–e); continuous irradiation was required for the formation of well‐defined, long helical nanoribbons. We did not detect important overheating due to LED irradiation (Table S5) or evaporation (5%). The molecular chirality of the monomer, defined by the single stereocenter of the phenylalanine, determined the helical sense of the supramolecular nanoribbon (Figure 3g) [44, 45]. Chiral nanoribbons display an averaged width of 29 nm, a tape thickness of 5.6 nm, a helical angle of 59°, and a helical pitch of 72 nm, as shown by the combination of EM and small‐angle x‐ray scattering (SAXS) (Figure 3h and S2). In addition, wide‐angle x‐ray scattering (WAXS) measurements confirmed short‐range molecular ordering within a bilayer‐like assembly, with first‐ and second‐order reflections at 4.48 and 9.08 nm^−1^, respectively (Figure 3i). These reflections correspond to a characteristic quasi‐periodic spacing of ca. 1.4 nm, which we tentatively associate with the packing of the hydrophobic domains. An overview of the geometrical features is shown in Table S6 and Figure S20. The average nanoribbon height, as measured by AFM, was fully consistent with the distances of short tapes observed by HRTEM (Figure S21).

The rate of photochemical reactions depends on parameters such as light intensity, emission wavelength, and the absorption path length [26]. Therefore, adjusting the **MCH** → **SP** conversion, either in continuous or discontinuous mode, should allow the fine‐tuning of the morphological transitions in the supramolecular system. We confirmed that steady‐state concentrations of **SP** could be precisely regulated by controlling the intensity and/or color of the irradiation source (Figure S22). This can be observed in the overall CD signal and in the sustained **MCH** concentration over time that can be achieved under fixed irradiation conditions (Figure S23). Increasing the **SP**/**MCH** ratio also yields increasing polymerization degrees, as seen from increased intensity of CD response (Figure 3j) [46]. As revealed by DLS experiments, simultaneous adjustment of the light‐emitting diodes (LEDs) duty cycle and emission range allowed the adjustment of the size of the nano‐assembly population (Figure 3i,j). Selection of polymorphs could also be observed in the structural features of the resulting nanoribbons by adjusting LED duty cycle and wavelength range (Figures 3k insert, and S23 and S24) [47]. Samples irradiated to yield lower **SP** concentrations showed less helical nanoribbons, higher pitch variance, and also higher width of a single ribbon; LEDs closer to the merocyanine absorption band generate shorter and more irregular helical nanoribbons. These observations confirm the key role of **MCH** → **SP** conversion rate to preserve long‐range helical order and sustain larger nanoribbon assemblies.

### 2.5 Rate Enhancement of Structural Evolution by Cycles of Irradiation and Dark Relaxation

**FIGURE 4:**
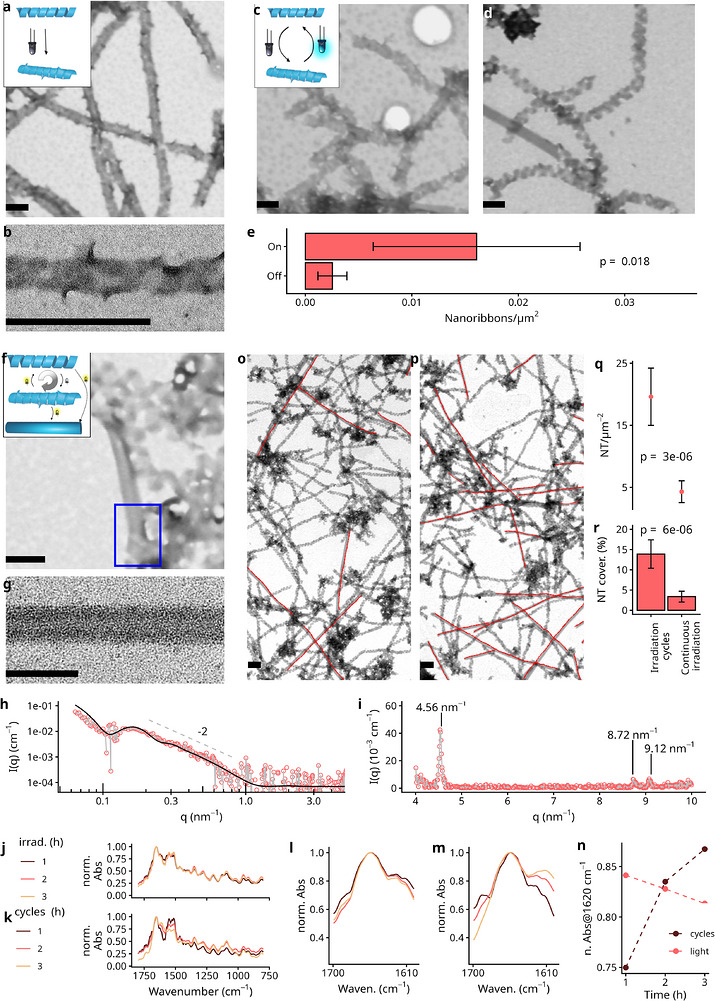
Assembly regulation by light. (a) Low magnification STEM micrographs showing the formation of collapsed helical nanoribbons after equilibration in the dark of helical nanoribbons. (b) High magnification TEM micrograph showing collapsed helical nanoribbons. (c) Reversibility of nanoribbon winding/unwinding after thermal relaxation. (d) After 3 h of visible light irradiation (broadband white light, 10 cd). (e) Nanoribbon number per area unit calculated for STEM micrographs before and after irradiation of collapsed nanoribbons. (f) Low STEM magnification, and (g) high magnification TEM micrographs of nanotubes formed during irradiation. Blue box shows a portion of unwound nanotube. (h) SAXS spectra, and (i) WAXS spectra of samples after reirradiation of a relaxed sample. (j) FTIR spectra of peptides assembled by continuous irradiation, and (k) over light‐dark cycles. (l, m) Magnification of the amide I region in continuously irradiated and cycled samples, respectively. Raw spectra are available in Figure S27. (n) Evolution of the absorbance at 1620 cm^−1^ in FTIR spectra in samples continuously irradiated or treated with alternated 30 min irradiation and thermal relaxation cycles (30 min of irradiation (broadband white light, 10 cd) and 30 min of relaxation in the dark). STEM micrographs showing nanotubes in samples: (o) continuously irradiated, and (p) irradiated in cycles of 30 min of irradiation (broadband white light, 10 cd) and 30 min of relaxation in the dark (500 µM, MES 50 mM pH 6, 45°C, 3 h). The red lines indicate the assemblies with nanotube morphology. (q) Quantification of changes in the nanotube density (1000 nanotubes/µm^2^), and (r) coverage. Scale bars denote 100 nm except in (o–p), where they denotes 500 nm.

Sufficiently long irradiation (3 h and more) with visible light, where the concentration of the **MCH** form is negligible, consistently yields a homogenous population of defined nanoribbons in solution (Figure 3c). Removal of the light source leads to the thermal back isomerization of the photoswitch toward the merocyanine form, causing the collapse of the helical nanoribbons (Figure 4a). The process starts by the exchange of the **SP** building blocks from the nanoassemblies to the bulk solution, where they undergo a spontaneous and fast isomerization from the **SP** to the thermally relaxed **MCH** state (Figure 2c). This process prevents effective self‐healing of the nanoribbons by the **MCH** monomers and creates void defects in the assembly that further accelerate monomer exchange and isomerization within the aqueous solution [48]. The resulting progressive strain imposed into the supramolecular system results in the nanoribbons collapse into distorted helical assemblies with a shorter helical pitch (Figure 4a,b). Application of a new light pulse to these collapsing nanoribbons formed upon thermal relaxation showed the full reversibility of the assembly process. STEM micrographs confirmed that the helicity could be recovered in re‐irradiated samples (Figures 4c,d and S25). However, along with the recovered original nanoribbons, a new emerging nanotubular species was identified in the light re‐irradiated samples (Figure 4f,g). Within a similar diameter range, the nanoribbons and the new nanotubular structures showed clear structural similarities. Although the coexistence of ribbons and nanotubes limits the unambiguous assignment of scattering data, the SAXS analysis of these cycled samples showed an increase in the mesoscopic ordering of the assemblies with an average thickness of 5.2 nm and diameter of 35 nm (Figure 4h). The nanotubular form displays a higher monodispersity compared to the nanoribbons, confirming a higher molecular ordering and also suggesting a more stable architecture. In addition, WAXS analysis of the self‐assembly process under cyclic light irradiation showed that the main scattering features are largely preserved, with only subtle shifts from 4.48 to 4.56 nm^−1^ and from 9.08 to 9.12 nm^−1^ relative to the nanoribbon pattern (Figure 4i). A new Bragg reflection also appears at 8.72 nm^−1^, corresponding to a d‐spacing of 0.72 nm, which may reflect a regular lateral organization of the peptide packing. These observations are consistent with the emergence of a more ordered nanoassembly under cyclic irradiation. Taken together, the higher monodispersity and internal molecular regularity validated the key role of the alternation in the light energy inputs to increase the internal order of the self‐assembling supramolecular system. It should also be noted that the electron microscopy detection of several specimens of hybrid tubular/helical lineal assemblies confirmed that nanotubular architectures were evolving from the collapsing nanoribbons that are enriched with more self‐assembling **SP** monomer after re‐illumination (Figures 4f and S26). Therefore, irradiation of the collapsed nanostructures follows two competing pathways: (i) recovery of the helical shape prior to the collapse or (ii) evolving toward the nanotubular architecture. These data can also be related to previous observations in perylenediimide systems where repeated chemical energy inputs biased the supramolecular nucleation/elongation pathway [38].

Following this observation, the system was exposed to oscillating light/dark periods (see Supporting Information). Dark cycles of half an hour were sufficiently long to yield a significant conversion of the photoswitch toward the merocyanine form— e.g., more than 75% of MCH — as calculated from the kinetic constant of the thermal relaxation (Figure S13). In the infrared spectra, the amide I region (1700–1600 cm^−1^) provides information on peptide folding and hydrogen bonding. Under continuous irradiation the spectra show contributions near 1650 and 1620 cm^−1^, whereas samples subjected to light/dark cycles display a relatively stronger contribution at ca. 1620 cm^−1^, consistent with increased *β*‐sheet‐like intermolecular organization under oscillatory light input. (Figure 4j–n), which indicated the formation of supramolecular structures with a higher contribution of peptide antiparallel *β*‐sheet folding under light input oscillations. This observation validates the molecular reorganization of the supramolecular structure during the light‐dark cycles and is consistent with the enhancement of the antiparallel *β*‐sheet ordering and the assembly rate following short periods of thermal relaxation (Figure 4n). The resulting faster proliferation of the nanotubular supramolecular structures when using oscillating irradiation compared to continuous light irradiation was also confirmed by STEM (Figure 4o). Thus, prolonged continuous irradiation can also lead to the evolution of the supramolecular system but does not reproduce the same degree of nanotube enrichment as cyclic driving. Differential scanning calorimetry (DSC) experiments on these samples showed that the transition temperature associated with the endothermic melting of the supramolecular structures increases from 65.8°C to 68.8°C in samples enriched with nanotubular structures, compared to those containing helical nanoribbons (Figure S28). These experiments validated the nanotubular polymorph as the thermodynamically favored state, while the helical nanoribbons are kinetically trapped intermediates sustained by the higher spiropyran to merocyanine mole ratio. Statistical analysis of the electron micrographs confirmed that the introduction of the dark cycles assisted the transition from the nanoribbons toward the more stable nanotubular structure, as compared to control samples irradiated continuously over the same time (Figure 4q,r). We found that an optimal production of nanotubular polymorphs required sufficient resting time provided by the dark phase. For example, shorter cycles (< 10 min irradiation, followed by an equivalent time dark period) were not sufficient to increase the production of nanotubular architectures (Figure S29). The newly formed tubular topology conserves the initial curvature and bending energy while reducing the interfacial tension of the corresponding helical precursor. This loss of interfacial energy supports the nanotubular polymorphs as thermodynamically more stable adducts compared to their helical counterparts [49]. Once the nanotube is formed, the lower strain of this polymorph stabilizes the tubular shape against deformations. Indeed, the density of nanotubes remains constant after light irradiation, indicating that nanotubes do not transition back to nanoribbons and confirming their higher stability during irradiation (Figure S30). However, the transient character of the nanotubes was confirmed by the progressive decrease of the nano‐assemblies' size distributions after dark periods (Figure S28). Further, STEM analysis validated the decrease of the nanotube length in the absence of light irradiation and that nanoribbons and tubes are eventually fully depleted (Figure S31).

The formation of nanotubes under cyclic irradiation can be rationalized as a kinetic pathway‐bias process rather than as simple reassembly from fully equilibrated monomers. During the dark period, **SP** → **MCH** thermal relaxation and monomer exchange occur faster than complete supramolecular disassembly, producing partially depleted, defect‐rich, collapsed nanoribbons. Upon renewed irradiation, the regenerated **SP** is reincorporated into these pre‐existing curved assemblies, which can either recover the helical nanoribbon morphology or evolve toward a closed tubular morphology. The latter pathway is favored upon repeated cycling because tube formation preserves the intrinsic curvature of the ribbon‐like precursor while reducing exposed lateral edges and interfacial energy. Thus, light/dark cycling repeatedly anneals the assemblies through a partially relaxed intermediate, allowing access to the more stable closed morphology that is kinetically less accessible under continuous irradiation. This interpretation is supported by the observation of collapsed nanoribbons and hybrid ribbon/tube intermediates, the increased nanotube density under cyclic compared with continuous irradiation, the enhanced *β*‐sheet‐like FTIR contribution under cyclic conditions, the higher monodispersity and internal order observed by SAXS/WAXS, and the higher DSC transition temperature of nanotube‐enriched samples. These observations support the assignment of the nanotubular polymorph as the more stable morphology under irradiation, whereas helical nanoribbons behave as kinetically accessible intermediates favored under continuous high‐**SP** conditions.

## 3 Conclusion

Dynamics of photoresponsive natural systems are inherently connected to discontinuity of energy consumption and diurnal cycles of solar irradiation. However, the influence of periodic discontinuity of energy supply over the structural transitions of synthetic self‐assembling systems has remained elusive. By looking at the effect of irradiation cycles on a self‐assembling peptide photoswitch, we have demonstrated adaptive responsiveness darkness‐driven structural self‐selection. Light irradiation transiently yields nanoribbons that in the absence of light energy collapse and ultimately disassemble. However, if the light supply is re‐established prior to the complete disassembly, the original nanoribbons can be recovered by the newly generated free spiropyran that repairs the structural defects. Following the collapse‐repair dynamics under light‐dark oscillations, a new transient tubular structure can emerge from the collapsing nanohelical supramolecular architectures. During the dark period, the supramolecular phase transition toward the nanotubular polymorph is assisted by this fluidification of the nanoribbon intermediates. Once formed, the more stable nanotubular structure does not transition back to the nanoribbons, however, still displays transient dynamics as shown by the shortening of the nanotubules and their full disassembly if kept away from the light supply for a prolonged time.

In summary, we here demonstrate that oscillating light irradiation can navigate the energy self‐assembling landscape of a supramolecular system through kinetically disfavored pathways and favor structural adaptation toward more stable polymorphs. These findings are of interest for stimuli‐responsive materials [50], whose physicochemical properties can be modified by enriching a specific self‐assembled polymorph under cyclic energy inputs. This study also suggests the connection between the cyclic and asymmetric nature of light energy and the increasing structural complexity developed by molecular and supramolecular photoresponsive networks. This work provides unique and solid evidence of structural selection under alternating energy supply for synthetic photosensitive supramolecular systems.

## Author Contributions

**Alejandro Méndez‐ardoy**: investigation, conceptualization, writing – original draft. **Nicolas Cissé**: investigation, writing – original draft. **Adrian Sanchez‐fernandez**: investigation, writing – original draft. **Patricia Fulias‐guzmán**: investigation, writing – original draft. **Marc C. A. Stuart**: investigation. **Tibor Kudernac**: conceptualization, funding acquisition, writing – original draft, supervision. **Javier Montenegro**: conceptualization, funding acquisition, writing – original draft, supervision.

## Conflicts of Interest

The authors declare no conflicts of interest.

## Supporting information



The authors have cited additional references within the Supporting Information [51, 52, 53, 54, 55, 56, 57, 58, 59, 60, 61, 62, 63].
**Supporting File**: anie72906‐sup‐0001‐SuppMat.pdf.

## Data Availability

The data that support the findings of this study are available from the corresponding author upon reasonable request.
