# Supplementary material for: Light and Dark Cycles Control the Structural Evolution of Photoresponsive Supramolecular Systems
Source: Angew Chem Int Ed Engl. 2026 Jun 2;65(31):e4843934. doi: 10.1002/anie.4843934 (PMC13411505; doi:10.1002/anie.4843934)
Supplement: Supplementary file 1 — The authors have cited additional references within the Supporting Information [51, 52, 53, 54, 55, 56, 57, 58, 59, 60, 61, 62, 63]. Supporting File: anie72906‐sup‐0001‐SuppMat.pdf. [file ANIE-65-e4843934-s001.pdf]

Supplementary Information for
Light and Dark Cycles Control the Structural Evolution of Photoresponsive Supramolecular Systems

Alejandro Méndez-Ardoy,[1] Nicolas Cissé,[2] Adrián Sanchez-Fernandez,[3] Patricia Fulias-Guzmán,[3] Marc C. Stuart,[2] Tibor Kudernac,[2,*] Javier Montenegro[3,*]

[1]Department of Organic Chemistry, Faculty of Chemistry, University of Seville, c/ Profesor García González 1, 41012 Sevilla, Spain; Instituto de Investigaciones Químicas (IIQ), CSIC Universidad de Sevilla, Avda. Américo Vespucio 49, 41092 Sevilla, Spain.
[2]Stratingh Institute for Chemistry, University of Groningen, Nijenborgh 4, 9747 AG Groningen, The Netherlands.
[3]Centro Singular de Investigación en Química Biolóxica e Materiais Moleculares (CIQUS), Departamento de Química Orgánica, Universidade de Santiago de Compostela, 15782 Santiago de Compostela, Spain.

Contents

S1 Experimental section **3**
- S1.1 Materials and methods 3
- S1.2 Synthesis of new derivatives 3
  - S1.2.1 Synthesis of 1 3
  - S1.2.2 Conjugation with the spiropyran moiety 4
  - S1.2.3 βAAG 5
  - S1.2.4 βAVG 5
  - S1.2.5 βAGF 5
  - S1.2.6 GFG 6
  - S1.2.7 βAFE 6
- S1.3 General methods 6
- S1.4 Abbreviations 13

S2 Supplementary figures **14**
- S2.1 Peptide sequence screening 14
- S2.2 Light Triggered Isomerization 17
- S2.3 Thermal Relaxation of the Photogenerated Spiropyran State 20
- S2.4 Self-Assembly of the Peptide Photoswitch 23
- S2.5 Formation of out-of-equilibrium Chiral Polymorphs by One Irradiation Cycle . . 26
- S2.6 Enhancement of Structural Evolution by Cycles of Irradiation and Dark Relaxation 32

S3 Characterization of new derivatives **37**
- S3.1 1 37
- S3.2 SP 39
- S3.3 SP (D) 41
- S3.4 βAAG 43
- S3.5 βAVG 44
- S3.6 βAGF 45
- S3.7 GFG 46
- S3.8 βAFE 47

S4 References **48**

S1 Experimental section

S1.1 Materials and methods

Reagents were acquired from Fluka, Aldrich, Iris Biotech or TCI. ^{1}H NMR was acquired in a Varian 300 MHz spectrometer. Spectra were normalized with respect the residual solvent signal (CD_3CN or DMSO-d_6) or water (D_2O). Analytical HPLC was carried out in an Agilent 1260 Infinity II equipped with an Agilent SB-C18 column and connected to a 6120 Quadrupole LCMS. HR-MS was acquired in a Bruker Microtof. Staining of STEM and TEM samples were carried out by using the commercial solutions of gadolinium (III) acetate tetrahydrate (Electron Microscopy Sciences). The spiropyran derivative **SP-CHO** was prepared as described in the literature.[51]

S1.2 Synthesis of new derivatives

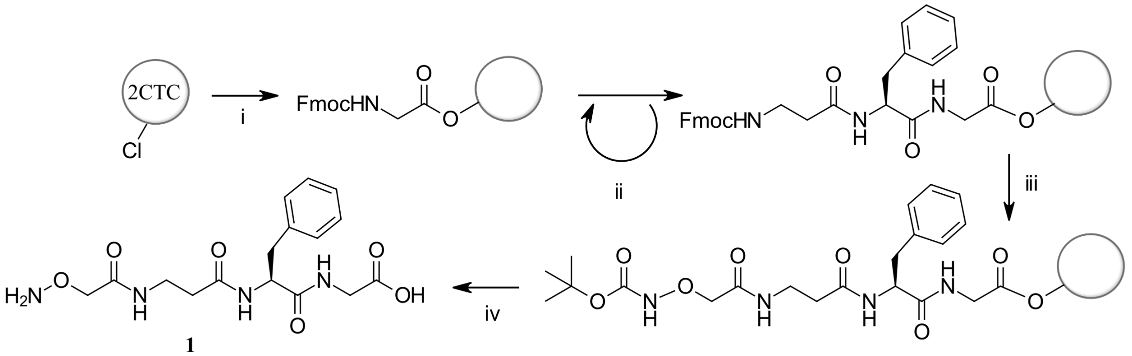

1

Scheme S1: Preparation of **1** by SPPS. Reagent and conditions: i) Fmoc-glycine, DIEA, DCM, 2 h; ii) piperidine/DMF (1:4), 10 min; then amino acid, *N*-HBTU, DIEA, DMF, 30 min, repeat 2 cycles with the corresponding amino acid; iii) piperidine/DMF (1:4), 10 min, then [(tert-butoxycarbonyl)aminoxy]acetic acid, *N*-HATU, DIEA, DMF, 45 min; iv) TFA-DCM-H_2O-TIS (9:0.5:0.25:0.25).

S1.2.1 Synthesis of 1

Peptides were prepared manually in solid phase as described in Scheme S1. 2-Chlorotrytil chloride resin (2CTC, 62.5 mg, 0.1 mmol, 1.6 mmol chloride/g resin) were soaked in freshly distilled DCM (3 mL) for 30 min. The solvent was filtered off, and a solution of Fmoc-Glycine (89.1 mg, 0.3 mmol, 3 equiv.) and DIEA (105 μL, 0.6 mmol, 6 equiv.) in freshly distilled DCM (3 mL) was added to the resin. After 2 h, the solvent was filtered off and the resin was washed with DCM (3× 3 mL). A mixture of DCM-MeOH-DIEA (8.5:1:0.5, 3 mL) was added and the resin was shaken for 30 min, filtered and finally successively washed with DCM (3 ×, 3 mL) and DMF (3× 3 mL).

The Fmoc group was removed by treatment with piperidine/DMF (1:4, 2-3 mL) for 10 min. The resin was washed with DMF (3× 3 mL) and then treated with a solution of Fmoc-protected amino acid (0.3 mmol), *N*-HBTU[52] (113 mg, 0.3 mmol) and DIEA (100 μL, 0.6 mmol) in DMF (3 mL). The resin was shaken for 20 min and then washed with DMF (3× 3 mL). Hereafter, a solution of (Boc-aminoxy)acetic acid (48 mg, 0.25 mmol) and *N*-HATU[52] (94 mg, 0.25 mmol) in DMF (1 mL) was added to the resin followed by the dropwise addition of DIEA (42 μL, 0.25

mmol) in DMF (1 mL). The resulting mixture was mechanically shaken for 45 min and finally, the resin was washed with DMF (3 ×, 3 mL).

The peptide was released from the resin by treatment with freshly prepared cleavage cocktail (TFA-DCM-H_2O-triisopropylsilane, 0.9:0.05:0.025:0.025, 3 mL) for 2 h and then filtered. The resin was washed with TFA (0.5 mL) and the combined acidic solutions were evaporated to 1-2 mL by bubbling argon. The concentrated solution was added dropwise to cold diethyl ether (10 mL diethyl ether/mL TFA). The resulting precipitate was centrifuged for 10 min at 3000 rpm. The supernatant was discarded, then fresh diethyl ether was added and the suspension was sonicated for 1 min and centrifuged. The sample was dried with a stream of air and dissolved in milliQ water and purified by semipreparative HPLC using a C18 column [gradient of H_2O-0.1% TFA-acetonitrile-0.1% TFA 95:5 (0 min) to 50:50 (20 min)] to give 8 mg of **1** (23 %); R_t = 7.09 min, RP-uHPLC (C18, H_2O-acetonitrile (dopped with 0.1% TFA) 95:5 (0 min) → 5:95 (15 min)); ^{1}**H NMR** (300 MHz, D_2O) δ: 7.32 (m, 5 H), 4.63 (dd, 1 H, J_9, $J_{6.0}$), 4.50 (s, 2 H), 3.93 (d, 2 H, $J_{2.3}$), 3.37 (m, 2 H), 3.14 (dd, 1 H, $J_{14.1}$, $J_{5.9}$), 2.93 (dd, 1 H, $J_{13.5}$, $J_{9.4}$), 2.43 (m, 2 H); **FTIR** (neat): 3292, 1645, 1539, 1422, 1185, 1131, 839, 798, 721, 697, 574, 496 cm^{-1}; **HRMS** calculated for $C_{16}H_{23}N_4O_6^+$: 367.1612, found: 367.1612.

Compound **1 (D)** was prepared in a similar fashion, but using Fmoc-D-phenylalanine. ^{1}**H NMR** (300 MHz, D_2O) δ: 7.23 (m, 5 H), 4.60 (dd, 1 H, $^3J_{H,H}$= 9.4 Hz, $^3J_{H,H}$ = 5.8 Hz), 4.45 (s, 2 H), 3.89 (d, 2 H, ^3J = 2.3 Hz), 3.34 (m, 2 H), 3.12 (dd, 1 H, $^2J_{H,H}$ = 14.7 Hz, $^3J_{H,H}$ = 5.9 Hz, 2.90 (dd, 1 H, $^2J_{H,H}$ = 13.5, $^3J_{H,H}$ = 9.4 Hz), 1.91 (m, 2 H). **HRMS** calculated for $C_{16}H_{23}N_4O_6^+$: 367.1612, found: 367.1611.

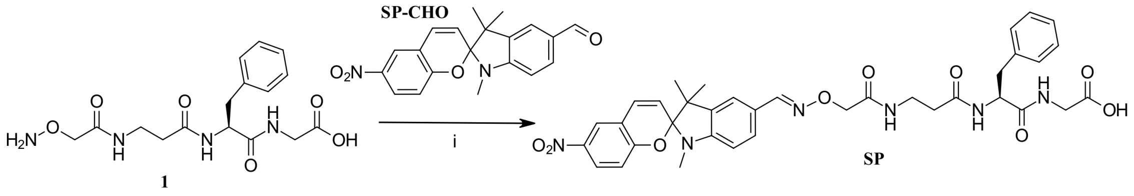

Scheme S2: Preparation of the peptide (**SP**) in solution. Reagent and conditions: i) ACN-DMSO, rt, 2h.

S1.2.2 Conjugation with the spiropyran moiety

Compound **SP** was prepared as described in Scheme S2: 10.5 mg of **1** (0.03 mmol) and **SP-CHO** (12 mg, 0.034 mmol, 1.1 equiv) were dissolved in 0.5 mL ACN containing 20 µL DMSO. The reaction was stirred at room temperature for 2 h. The mixture was diluted with H_2O-ACN 1:1 and purified by preparative HPLC using a C18 column [gradient of H_2O-0.1% TFA-acetonitrile-0.1% TFA 70:30 (0 min) to 5:95 (20 min)] to give 8 mg of **SP** (38 %); R_t = 12.36 min, RP-uHPLC (C18, H_2O-acetonitrile (dopped with 0.1% TFA) 95:5 (0 min) → 5:95 (15 min)); ^{1}**H NMR** (300 MHz, CD_3CN-D_2O 2:1) δ: 8.17 (s, 1 H), 8.05 (d, 1 H, $^4J_{H,H}$= 2.3 Hz), 7.98 (dd, 1 H, $^4J_{H,H}$ = 2.9 Hz, $^3J_{H,H}$ = 9.0 Hz), 7.35 (m, 2 H), 7.20 (m, 5 H), 7.04 (d, 1 H, $^2J_{H,H}$ = 10.6 Hz), 6.68 (d, 1 H, $^3J_{H,H}$ = 8.8 Hz), 6.58 (d, 1 H, $^3J_{H,H}$ = 8.2 Hz), 5.90 (d, 1 H, $^2J_{H,H}$ = 10.5 Hz), 4.52 (m, 1 H), 4.44 (s, 2 H), 3.82 (d, 2 H, $^3J_{H,H}$ = 5.3 Hz), 3.09, 3.04 (dd, 1 H, $^3J_{5.3}$, $^2J_{15}$), 2.76 (m, 1 H), 2.71 (s, 2 H), 2.30 (m, 2 H), 1.15 (bs, 6 H); ^{13}**C NMR** (125.7 MHz, DMSO-d_6) δ: 172.1, 171.5, 170.8, 169.4, 159.6, 151.3, 149.8, 141.1, 138.4, 137.3, 129.6, 129.0, 128.5, 126.7, 126.3, 123.3, 123.2, 121.4,

120.0, 119.3, 115.9, 107.4, 106.4, 92.4, 72.9, 54.0, 52.2, 43.0, 41.1, 41.0, 37.9, 35.5, 35.3, 28.9, 25.9, 20.0 **FTIR** (neat): 3292, 2929, 1755, 1617, 1522, 1338, 1271, 1161, 1083, 1017, 952, 917, 805, 747, 701, 572 cm^{-1}; **HRMS** calculated for $C_{36}H_{39}N_6O_9^+$: 699.2773, found: 699.2774.

Derivative **PS (D)** was prepared in the same way, but using **1 (D)** instead **1**: R_t = 12.5 min, RP-uHPLC (C18, H_2O-acetonitrile (dopped with 0.1% TFA) 95:5 (0 min) → 5:95 (15 min)); ^{1}**H NMR** (300 MHz, CD_3CN (few drops of D_2O)) δ: 8.18 (s, 1 H), 8.07 (d, 1 H, $^4J_{H,H}$ = 2.8 Hz), 7.99 (dd, 1H, $^4J_{H,H}$ = 2.8 Hz, $^3J_{H,H}$ = 9.1 Hz), 7.37 (m, 2 H), 7.22 (m, 5 H), 7.06 (d, 1 H, $^2J_{H,H}$ = 10.4 Hz), 6.71 (d, 1 H, $^3J_{H,H}$ = 8.9 Hz), 6.60 (d, 1 H, $^3J_{H,H}$ = 8.1 Hz), 5.92 (d, 1 H, $^3J_{H,H}$ = 5.9 Hz), 4.53 (m, 1 H), 4.46 (s, 2 H), 3.82 (m, 2 H), 3.09 (dd, 1 H, $^3J_{H,H}$ = 5.2 Hz, $^2J_{H,H}$ = 14.0 Hz), 2.76 (m, 4 H), 2.71 (s, 2 H), 2.31 (m, 2 H), 1.26 (s, 3 H), 1.13 (s, 3 H); **HRMS** calculated for $C_{36}H_{39}N_6O_9^+$: 699.2773, found: 699.2773.

S1.2.3 βAAG

The peptide was prepared as described in the experimental section with an overall yield of 2% R_t = 11.60 min, RP-uHPLC (C18, H_2O-acetonitrile (dopped with 0.1% TFA) 95:5 (0 min) → 5:95 (15 min)); ^{1}**H NMR** (500 MHz, D_2O-CD_3CN 1:1) δ: 8.55 (s, 1 H), 8.41 (d, 1 H, $^4J_{H,H}$ = 2.8 Hz), 8.35 (dd, 1 H, $^3J_{H,H}$ = 9.0 Hz, $^4J_{H,H}$ = 2.8 Hz), 7.74 (s, 1 H), 7.71 (d, 1 H, $^3J_{H,H}$ = 8.2 Hz), 7.34 (d, 1 H, $^3J_{H,H}$ = 10.5 Hz), 7.06 (d, 1 H, $^3J_{H,H}$ = 9.1 Hz), 6.97 (d, 1 H, $^3J_{H,H}$ = 8.1 Hz), 6.68 (d, 1 H, $^3J_{H,H}$ = 10.4 Hz), 4.88 (s, 2 H), 4.52 (q, 1 H, $^3J_{H,H}$ = 7.2 Hz), 4.16 (s, 2 H), 3.78 (t, 2 H, $^3J_{H,H}$ = 6.5 Hz), 3.09 (s, 3 H), 2.77 (t, 2 H, $^3J_{H,H}$ = 6.5 Hz), 2.23 (s, 1 H), 1.52 (m, 8 H); **HRMS** calculated for $C_{30}H_{33}N_6O_9^-$: 621.2309, found: 621.2292.

S1.2.4 βAVG

The peptide was prepared as described in the experimental section with an overall yield of 3% R_t = 11.86 min, RP-uHPLC (C18, H_2O-acetonitrile (dopped with 0.1% TFA) 95:5 (0 min) → 5:95 (15 min)); ^{1}**H NMR** (500 MHz, D_2O-CD_3CN 1:1) δ: 8.69 (s, 1 H), 8.57 (d, 1 H, $^4J_{H,H}$ = 2.9 Hz), 8.50 (dd, 1 H, $^3J_{H,H}$ = 9.1 Hz, $^4J_{H,H}$ = 3.0 Hz), 7.89 (s, 1 H), 7.86 (d, 1 H, $^3J_{H,H}$ = 8.3 Hz), 7.55 (d, 1 H, $^3J_{H,H}$ = 10.5 Hz), 7.21 (d, 1 H, $^3J_{H,H}$ = 9.1 Hz), 7.11 (d, 1 H, $^3J_{H,H}$ = 8.1 Hz), 6.42 (d, 1 H, $^3J_{H,H}$ = 10.4 Hz), 5.00 (s, 2 H), 4.58 (d, 1 H, $^3J_{H,H}$ = 7.0 Hz), 4.34 (m, 2 H), 3.93 (t, 2 H, $^3J_{H,H}$ = 6.9 Hz), 3.22 (s, 3 H), 2.96 (m, 2 H), 1.72 (m, 5 H), 1.62 (m, 3 H), 1.32 (m, 6 H). **HRMS** calculated for $C_{32}H_{37}N_6O_9^-$: 649.2622, found: 649.2617.

S1.2.5 βAGF

The peptide was prepared as described in the experimental section with an overall yield of 2% R_t = 12.41 min, RP-uHPLC (C18, H_2O-acetonitrile (dopped with 0.1% TFA) 95:5 (0 min) → 5:95 (15 min)); ^{1}**H NMR** (500 MHz, D_2O-CD_3CN 1:1) δ: 8.62 (s, 1 H), 8.50 (d, 1 H, $^4J_{H,H}$ = 2.8 Hz), 8.41 (dd, 1 H, $^3J_{H,H}$ = 9.0 Hz, $^4J_{H,H}$ = 2.8 Hz), 7.89 (s, 1 H), 7.77 (d, 1 H, $^3J_{H,H}$ = 8.1 Hz), 7.61 (m, 5 H), 7.49 (d, 1 H, $^3J_{H,H}$ = 10.4 Hz), 7.10 (d, 1 H, $^3J_{H,H}$ = 9.0 Hz), 7.03 (d, 1 H, $^3J_{H,H}$ = 8.1 Hz), 6.34 (d, 1 H, $^3J_{H,H}$ = 10.4 Hz), 4.96 (m, 3 H), 4.04 (m, 2 H), 3.83 (t, 2 H, $^3J_{H,H}$ = 6.5 Hz), 3.51 (dd, 1 H, $^2J_{H,H}$ = 14.1 Hz, $^3J_{H,H}$ = 5.3 Hz), 3.35 (dd, 1 H, $^2J_{H,H}$ = 14.0 Hz, $^3J_{H,H}$ = 8.2 Hz), 2.83 (t, 2 H, $^3J_{H,H}$ = 6.5 Hz), 1.69 (m, 6 H); **HRMS** calculated for $C_{36}H_{37}N_6O_9^-$: 697.2622, found: 697.2620.

S1.2.6 GFG

The peptide was prepared as described in the experimental section with an overall yield of 2% R_t = 12.69 min, RP-uHPLC (C18, H_2O-acetonitrile (dopped with 0.1% TFA) 95:5 (0 min) → 5:95 (15 min)); ^{1}**H NMR** (500 MHz, D_2O-CD_3CN 1:1) δ: 8.68 (s, 1 H), 8.52 (b, 1 H), 8.43 (bd, 1 H, $^3J_{H,H}$ = 9.0 Hz), 7.88 (s, 1 H), 7.81 (d, 1 H, $^3J_{H,H}$ = 8.0 Hz), 7.61 (m, 5 H), 7.48 (d, 1 H, $^3J_{H,H}$ = 10.4 Hz), 7.08 (m, 2 H) 6.35 (d, 1 H, $^3J_{H,H}$ = 10.6 Hz), 4.99 (m, 2 H), 4.24 (m, 2 H), 3.51 (dd, 1 H, $^2J_{H,H}$ = 13.7 Hz, $^3J_{H,H}$ = 4.5 Hz), 3.35 (m, 1 H), 3.16 (s, 3 H), 2.58 (m, 2 H), 1.94 (m, 2 H), 1.69 (bs, 3 H), 1.50 (bs, 3 H); **HRMS** calculated for $C_{35}H_{35}N_6O_9^-$: 683.2466, found: 683.2463.

S1.2.7 βAFE

The peptide was prepared as described in the experimental section with an overall yield of 10% R_t = 12.69 min, RP-uHPLC (C18, H_2O-acetonitrile (dopped with 0.1% TFA) 95:5 (0 min) → 5:95 (15 min)); ^{1}**H NMR** (500 MHz, CD_3CN 1:1) δ: 8.16 (s, 1 H), 8.03 (d, 1 H, $^4J_{H,H}$ = 2.8 Hz), 7.95 (dd, 1 H, $^3J_{H,H}$ = 9.1 Hz, $^4J_{H,H}$ = 2.8 Hz), 7.37 (s, 1 H), 7.33 (d, 1 H, $^3J_{H,H}$ = 8.2 Hz), 7.20 (m, 2 H), 7.14 (m, 3 H), 7.02 (d, 1 H, $^3J_{H,H}$ = 10.4 Hz), 6.65 (m, 1 H), 6.57 (d, 1 H, $^3J_{H,H}$ = 8.1 Hz), 5.88 (d, 1 H, $^3J_{H,H}$) 10.4 Hz), 4.51 (m, 1 H), 4.44 (s, 2 H), 3.81 (m, 2 H), 3.28 (m, 2 H), 3.04 (dd, 1 H, $^2J_{H,H}$ = 14.2 Hz, $^3J_{H,H}$ = 5.4 Hz), 2.74 (m, 1 H), 2.69 (s, 3 H), 2.31 (m, 2 H), 1.12 (s, 3 H), 1.10 (s, 3 H); **HRMS** calculated for $C_{39}H_{43}N_6O_{11}^-$: 771.2984, found: 771.2955.

S1.3 General methods

Preparation of peptide solutions For experiments starting with very low preassembled state, approximately 1 mg of solid **SP** was sonicated in the presence of 2 mL MES 20 mM pH 6 at 45 °C for 17 min. After cooling down, the solution was filtered through 0.2 µm syringe filters. Concentration of the sample was determined by dissolving a small volume (10 µL) in water-ACN (1:1, 490 µL) and comparing the absorbance to a previously prepared calibration curve. After sample preparation, the mother solution was aliquoted and stored in the freezer (-30 °C) and used over a period of a week. Dilutions of the mother solution were carried out always with MES 20 mM pH 6 filtered through a 0.2 µm syringe filter. Diluted samples were heated at 50 °for 4 min and allowed to cool down in the dark. For experiments where higher concentrations were required, the filtering step was skipped. Instead, a small amount of the peptide (0.5-1 mg) was dissolved in 30 µL of a mixture HFIP-water 1:1, and the solution was heated at 45 °C for 20 min in a sealed 1.5 mL eppendorf, then briefly centrifuged. The solution was diluted to 1.5 mL MES buffer 50 mM pH 6, and the solution was centrifuged in the dark. The concentration was measured by UV readings (see above). The ionic strength was estimated to be 50 mM based on the buffer concentration employed.

Calibration curve A calibration curve for the determination of **SP** concentration by UV-Vis spectroscopy in water-ACN 1:1 was prepared. Dilutions of a 50 µM stock solution were prepared, then these samples were irradiated until complete decoloration and full vanishing of the merocyanine band located at ca 515 nm. The UV-Vis spectra was acquired and concentrations were plotted against absorbances at 320 nm.

Light sources and continuous/cycled irradiation experiments The light source was selected according the experimental conditions. Single-pulse light irradiations were carried out using a broadband white light LED (38 cd, 3 W) at a distance of *ca* 1 mm. In these conditions, complete decoloration was achieved in less than 2 seconds. Alternatively, experiments where continuous irradiation at fixed temperature, variable light intensity, or pulse programs were required, irradiation was carried out with white LED (10 cd, 105 mW) at a distance inferior to 1 cm. Irradiation under different wavelengths was carried out using commercial LEDs: green (20 cd, λ= 525 nm), orange (7 cd, λ= 590 nm) or red (10 cd, λ= 629 nm).

In all cases, in order to precisely regulate light intensity and irradiation times, LEDs were controlled using an Arduino One board. Except for 3 W LED, individual LEDs were connected to the digital output of the controller in series with 220 Ω resistances. Pulse Width Modulation (PWM) was used to regulate the amount of light per unit of time: briefly, PWM varies the voltage applied to the digital output (off, 0 V or on, 5 V) in adjustable pulse sequences, where higher duty cycles turn on the led for longer overall periods than for lower duty cycles.[53] For the 3 W LED, an external battery was used as power supply, and the LED was interfaced with the controller through a MOSFET.

For experiments under continuous light irradiation, samples were equilibrated at fixed temperatures in a Biosan TS-C100 ThermoShakers or LLG Uniblocktherm. 0.5 or 1.5 mL eppendorf caps were perforated in order to insert the LED cathode and anode.

Circular dichroism experiments Circular dichroism spectra were acquired in a Jasco J-1100 CD spectrometer which holder temperature was controlled through a peltier. Unless otherwise indicated, measurements were carried out in an 2 mm light path quartz cuvette. The solvent background signal was acquired and used as baseline. The following parameters were used during the acquisition: band width = 1 nm; response = 2 sec; scanning speed = 200 nm/min; data pitch = 0.2 nm; number of accumulations = 1.

Dynamic Light Scattering (DLS) DLS measurements were carried out in a Malvern Zetasizer Nano. All buffers used for dilution were filtered through a 0.2 µm syringe filter. Measurements were carried out at a fixed temperature of 20, 25, 30, 35 or 45 °C. In time-dependent assembly experiments, the attenuator was adjusted according the evolution of light scattering, in order to prevent nonlinear response above 1000 kcounts.

Small and wide angle X-ray scattering (SAXS and WAXS) SAXS and WAXS measurements were performed on a SAXSpoint 5.0 (Anton Paar) equipped with a Cu microsource (λ = 0.154 nm). Data were acquired at 1608 mm and 500 mm sample-to-detector distances for the SAXS and WAXS configurations, respectively. For SAXS measurements, samples in solution were loaded in 1.5-mm pathlength quartz capillaries. Samples deposited in Kapton windows were used for WAXS measurements. All measurements were performed at 25 °C using temperature-controlled sample environments and the concentration of peptide was 1 mM. A white light source inside the instrument ensured the illumination of the samples during data acquisition. Samples were exposed to the beam for 30 seconds/frame and data were accumulated for 120 frames. No radiation damage was observed with the used acquisition times. Raw data were reduced and averaged using the standard protocols of the instrument to yield the scattering intensity in absolute scale (*I(q)* in cm^{-1}

vs q in nm^{-1}). The signals from the buffer and capillary (in SAXS measurements) or the Kapton windows (in WAXS measurements) were subtracted accordingly.

The SAXS profiles exhibited pronounced oscillations at low q, which are characteristic consistent with the form factor of hollow cylindrical objects and can indicate well-defined geometrical features. Accordingly, data were analysed using a hollow cylinder form factor, which directly captures these shape-specific scattering features and allows extraction of physically meaningful parameters. Alternative models were considered but found to be less appropriate. A lamellar model was discarded because, although the WAXS data indicate a lamellar periodicity at the molecular scale, it does not reproduce the oscillatory behaviour observed in the SAXS regime and therefore cannot account for the overall tubular morphology of the assemblies. Likewise, the Beaucage model was not employed, as a description based on Guinier and Porod regimes would not capture the interference oscillations arising from the tubular geometry and would therefore not yield direct structural parameters. Taken together, these considerations justify the use of a hollow cylinder model as the most physically meaningful description of the form factor of the scatterers.

SAXS data were fitted using a model-based approach implemented in the SASview 5.0 suite. Considering the structure observed by EM, a hollow tube model was selected as the most appropriate to analyze the data. This model is parametrized by the thickness of the tape, the diameter of the assembly and its length (see Figure S1). In addition, a polydispersity function (PDI, Schulz distribution) was used for the radius of the structure to account for subtle deviations from a monodisperse assembly.

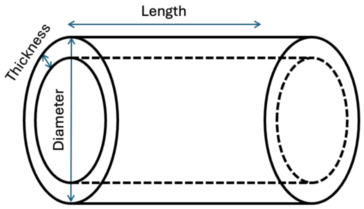

Figure S1: Schematic representation of the hollow cylinder described by the mathematical model used to analyze the data.

The instrument in SAXS mode was configured to access information on the cross-sectional structure of the assemblies. As such, the length or the helical pitch of the assemblies (> 1 μm and 72 nm, respectively) could not be determined using this technique as these are beyond the experimental resolution. However, this method complements the microscopy results by elucidating detailed structural features of the short dimensions that are hardly accessible by STEM. The results from the analysis of SAXS data are presented in Table S1:

Structure	Tape thickness (nm)	Diameter (nm)	Length (nm)	PDI
Helices	5.6 ± 0.2	41 ± 4	> 100	0.25
Tubes	5.2 ± 0.2	35 ± 2	> 100	0.1

Table S1: Structural features of helices and tubes.

WAXS data were analyzed by extracting the position of the peaks in the q scattering vector and converted to real-space dimensions using Bragg's Law:

$$d = \frac{2\pi}{q} \tag{S1}$$

Differential scanning calorimetry (DSC) The thermal stability of the assembled structures was determined using a DSC 200 (TA Instruments). Samples were prepared at a concentration of 500 μM and 5 μL aliquots were loaded in Tzero aluminum pans (TA Instruments). The excess molar heat capacity (C_p) was measured at a scan rate of 10 °C/min in the temperature range between 50 °C and 80 °C. The characteristic transition temperature of the supramolecular structures (T_m) was determined by the maximum heat capacity at the transition.

UV spectra measurements UV measurements were carried out in a Cary Varian 500 which holder temperature was regulated through a peltier. Unless otherwise indicated, measurements were carried out in a 0.2 cm quartz cuvette.

Infrared experiments A solution 0.5 mM was prepared at pH 6 by addition of small aliquots of diluted NaOH. The sample was either continuously irradiated or irradiated in cycles. At given intervals, 10 ţL of the sample were placed in the ATR and a film was formed using a stream of nitrogen. Infrared spectra were acquired in a Bruker Tensor 27 equiped withy an ATR with 128 scans acquisitions. Because the film formation is uneven, we normalized the corresponding signal in the manuscript Figure.

Nanoparticle tracking analysis experiments Buffers were filtered through a 0.45 μm syringe filters. In order to monitor in real time the changes in concentration of nanoagreggates, time-dependent NTA experiments were carried out a temperature of 20 °C. Nanoaggregates were observed under a NanoSight NS300 (Malvern Instruments Ktd). Approximately 1 mL of sample of concentration 130 μM in MES 20 mM pH 6 was irradiated and introduced by the microfluidic pump. Every 5 min, 3 videos were acquired, and nanoaggregate concentrations and size distributions were averaged. After each measurement set, the cell was renewed from the 1 mL stock.

Seeded experiments A portion of the solution was irradiated with visible light at room temperature until complete decoloration and allowed to equilibrate for 30 min. Assembly of the seed was assessed by CD. Then, defined volumes were mixed with non-irradiated solutions, irradiated and the assembly monitored by CD.

Spiropyran-merocyanine isomerization experiments In order to estimate the thermal merocyanine to spiropyran conversion, preassembled samples (110 μM in MES buffer 50 mM pH 6) were irradiated with visible light and then transferred to a cuvette (0.2 cm path length). The evolution of the merocyanine formation was monitored by reading the absorbance at 515. Temperatures of 20, 30, 35 and 45 °C were studied.

Observed kinetic constants corresponding the thermal isomerization from the spiropyran towards merocyanine isomer were obtained from UV-visible kinetic experiments, as described before (see reference 40 from the manuscript):

$$A_t = A_\infty - [A_\infty - A_0] \times \exp(-k_{obsd} t) \tag{S2}$$

where A_t is the absorbance of the merocyanine isomer at time t, A_∞ is the absorbance when $t \to \infty$, A_0 is the absorbance at $t = 0$ and k_{obsd} is the observed kinetic constant which contains the sum of the forward and reverse isomerization constants.

Determination of the supramolecular polymerization thermodynamic constants The polymerization was studied by UV measurements. Dilutions of the peptide ranging from 600-1 μM concentrations in MES 50 mM pH 6 were equilibrated for at least 2 h at room temperature under continuous light irradiation. UV spectra were acquired at room temperature using 1, 0.2 and 0.1 cm path length cuvettes using MES 50 mM as blank. The data was fitting to a isodesmic polymerization model adapted to the analytical expressions described in the literature [54]:

$$R_{295/320} = \frac{2Kc + 1 - \sqrt{4Kc+1}}{2K^2c^2}(R_{mon} - R_{pol}) + R_{pol} \tag{S3}$$

Where $R_{295/320}$ is the observed absorbance ratio at 295 and 320 nm, R_{mon} denotes the ratio for the monomer, R_{pol} denotes the ratio for the polymerized form, K is the polymerization thermodynamic constant, and c is the total **SP** concentration.

Determination of the initial merocyanine content In order to estimate the amount of **MCH** before assembly experiments, HPLC chromatograms were acquired in the presence of 3-methyl nitrobenzoic acid as internal standard. 40 μL **SP** stock solution were diluted to 500 μL water-ACN containing 3-methyl nitrobenzoic acid 0.5 mM and kept and injected at dark. After acquiring the corresponding chromatogram, the sample was irradiated and injected again. The percentage of **MCH** was estimated from the following relationship:

$$\%MCH = \left(1 - \frac{\frac{A_{SP(SP),1}}{A_{MNBA,1}}}{\frac{A_{SP(SP),2}}{A_{MNBA,2}}}\right) \times 100 \tag{S4}$$

where $A_{SP(SP),1}$, $A_{MNBA,1}$and $A_{SP(SP),2}$, $A_{MNBA,2}$ are the areas under the chromatogram for **SP** and 3-methyl nitrobenzoic acid in the first and second injection respectively. Because no traces or **SP** were observed after irradiation, we assume that the differences in **SP** are due to **MCH** → **SP** conversion.

Preparation of samples for electron microscopy Cu grids (400 mesh, coated with carbon grid, Ted Pella) were used to deposit the samples at concentration indicated. In each experiment, a 10-15 μL droplet of the sample was deposited on parafilm, and the grid was placed on top of the droplet through the shiny side for 2 min. Then the sample is placed through the dull side in filter paper to remove excess of solution and allowed to dry for a few hours. Next, samples were stained by deposition in 15 μL gadolinium (III) acetate tetrahydrate solution droplets[55] diluted according the manufacturer (1:4 dilution in milliQ water) for 10 min, then washed thoroughly with milliQ water (ca 400-500 μL) and dried over filter paper overnight. Image analysis was carried out manually using ImageJ.[56, 7] A detailed description of the procedure for the analysis of structures, including nanoribbons and nanotubes is described in the corresponding paragraph below.

STEM measurements STEM images were acquired in a Zeiss Ultra Plus scanning transmission electron microscope operating at an extra high tension of 20 kV.

HR-TEM measurements Micrographs were acquired in a Zeiss Libra 200 operating at 120 kV.

Cryo-TEM measurements The cryo-TEM samples were prepared by depositing a few microliters of these solutions on carbon-coated grids (Quantifoil 3.5/1, Quantifoil Micro Tools, Jena, Germany). After blotting the excess liquid, the grids were vitrified in liquid ethane (Vitrobot, FEI, Eindhoven, The Netherlands) and transferred to a Philips CM 120 microscope equipped with a Gatan model 626 cryo-stage operating at 120 kV. Micrographs were recorded under low-dose conditions with a slow-scan CCD camera. The highest density is obtained from the electron dense aromatic part of the building blocks

AFM Atomic force microscopy measurements were carried out in a Park Systems NX-10. ACTA tips were used (silicon tips, nominal values: spring constant = 40 N/m, frequency = 300 kHz, ROC less than 10 nm). Aqueous samples of peptides (pH ca 8-9) (15-20 µL, 1.8 mM) were deposited in freshly cleaved mica. After 15-20 min equilibration, the micas were thoroughly rinsed with milliQ water and dried with a steam of Ar, then measured immediately. Image analysis was carried out with Gwyddion.[58] Typically, the profiles were leveled by using the mean plane substraction. Tip strokes were reduced by aligning rows using the mean.

Determination of the autocatalyitic kinetic constants The kinetic constants for nucleation and growth were estimated by using a minimal autocatalytic model,[59] which has been to study the protein aggregation kinetic data (see reference 39 from manuscript) and autocatalytic formation of perylenediimides supramolecular assemblies formed in the presence of chemical fuels (see reference 38 from manuscript). The model proposes the following mechanism:

$$\mathrm{A} \xrightarrow{\mathrm{k_1}} \mathrm{B}$$
$$\mathrm{A + B} \xrightarrow{\mathrm{k_2}} 2\,\mathrm{B}$$

where k_1 and k_2 are the nucleation and growth kinetic constants respectively. The rate equations can be solved analytically and normalized o give:

$$[\mathrm{B}] = \left([\mathrm{A}]_0 - \frac{\frac{k_1}{k_2} + [\mathrm{A}]_0}{1 + \frac{k_1}{k_2[\mathrm{A}]_0} \times \exp\left(k_1 + k_2[\mathrm{A}]_0\right)t} \right) / [\mathrm{A}]_0 \tag{S5}$$

CD and DLS data was were fitted using the following general expression assuming single and linear contribution of [B] to the analytical response:

$$R = \alpha \times [\mathrm{B}] + R_i \tag{S6}$$

Being R the response on the detector (CD signal, cont rates), α a response proportionality constant, and R_i the response at $t = 0$, both considered as fitting parameters.

Data analysis Data analysis was carried out in R[60] and packages ggplot2,[61] nlstools[62] and minpack.lm[63]

Structural characterization of nanoribbons

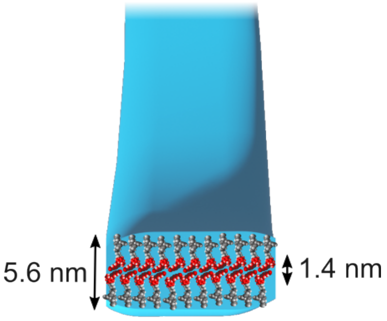

Figure S2: Assignment SAXS measurements.

Analysis of helical nanoribbons

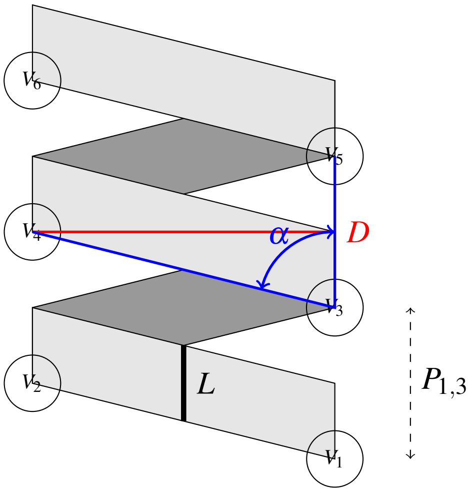

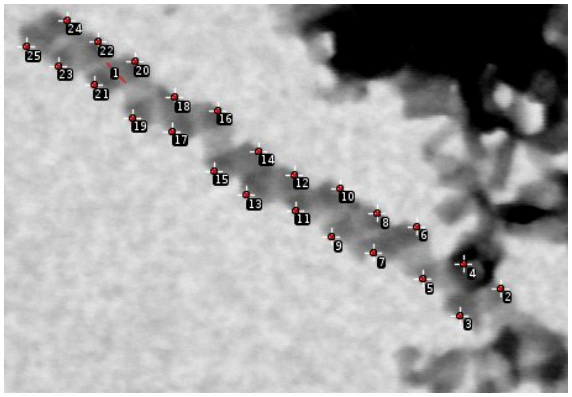

In order to extract statistical information concerning the structural parameters of nanoribbons, the TEM and STEM projections of ribbons were modeled as a set of vertex corresponding to the edges of the ribbon, V_i where i = 1,2...,6). The pitch for complete turns are therefore defined by the segments connecting V_i and V_{i+2}. The pitch angle (α) was calculated from the V_{i+1}, V_i, V_{i+2} angle, where V_i is the vertex. The ribbon diameter (D) was calculated from the pitch angel and the distance V_i to V_{i+1} considering $D = sin\alpha \times L_{V_i,V_{i+1}}$ Additionally, the thickness of the tape (L) was also measured in a well-visible location.

The edges of visible ribbons were marked with ImageJ, and the Cartesian coordinates for each node were stored for individual ribbons. We used a homemade R script in order to extract the euclidean distance and compute the averaged values.

Determination of nanotube coverage All tube structures found in micrographs were measured manually using a segmented line in order to correct tube curvatures. Additionally, the percentage of organic material in each micrograph was determined by adjusting the image threshold

and measuring the area fraction covered by the nanostructures in the binary images. The following parameters were calculated from individual micrographs:

$$Density = \frac{n}{A} \tag{S7}$$

and

$$Coverage = \frac{\overline{D} \times \sum_{i=1}^{n} L_i}{A} \times 100 \tag{S8}$$

Where A is the covered surface area, $\overline{D}$ is the average nanotube diameter, L_i is the length of tube i, and n is the number of individual tubes

S1.4 Abbreviations

DCM: Dichloromethane, HBTU: (2-(1*H*-benzotriazol-1-yl)-1,1,3,3-tetramethyluronium hexafluorophosphate HFIP: 1,1,1,3,3,3-Hexafluoro-2-propanol, HATU: *N*-(Dimethylamino)-1*H*-1,2,3-triazolo-4,5-*b*pyridin-1-ylmethylene-*N*-methylmethanaminium hexafluorophosphate *N*-oxide

S2 Supplementary figures

S2.1 Peptide sequence screening

Entry	Peptide sequence	Assembled morphology
1	βAFG	helical nanoribbons
2	βAAG	sheet-like/nanoribbons
3	βAVG	sheet-like
4	βAGF	amorphous
5	βGFG	amorphous
6	βAFE	thin helical nanoribbons

Table S2: Peptide mutants prepared in this work. The spiropyran was attached to the N terminus in all cases through a β-alanine linker.

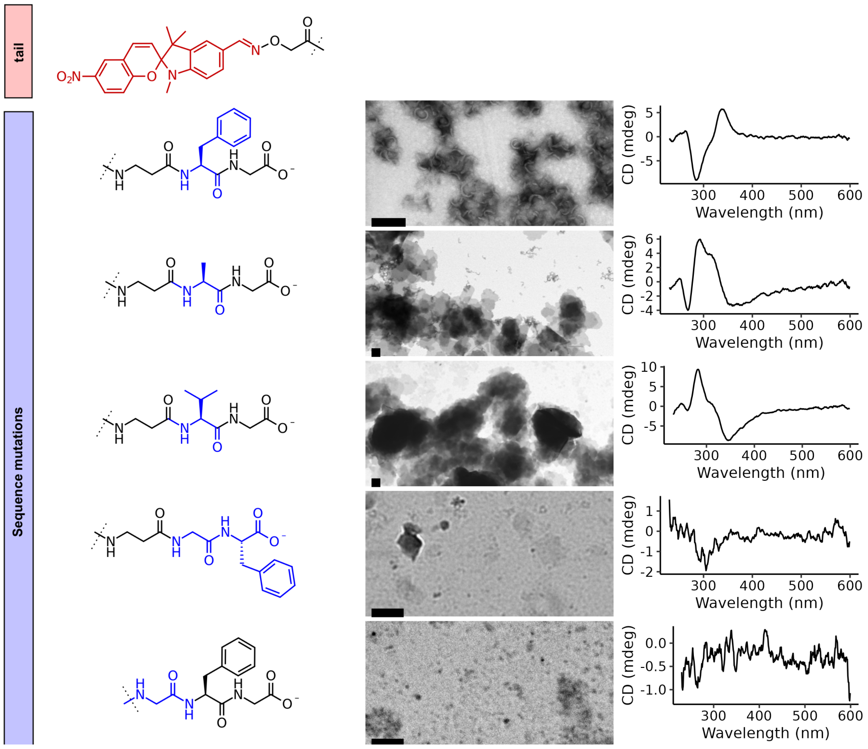

Figure S3: Effect of the peptide sequence on the supramolecular assembly capabilities of spiropyran-functionalized peptides. Samples were assembled at a concentration of 125 μM in MES 20 mM pH 6. Center, TEM micrographs after negative staining (center, scale bars are 200 nm or 1000 nm (GFG); right, circular dichroism.

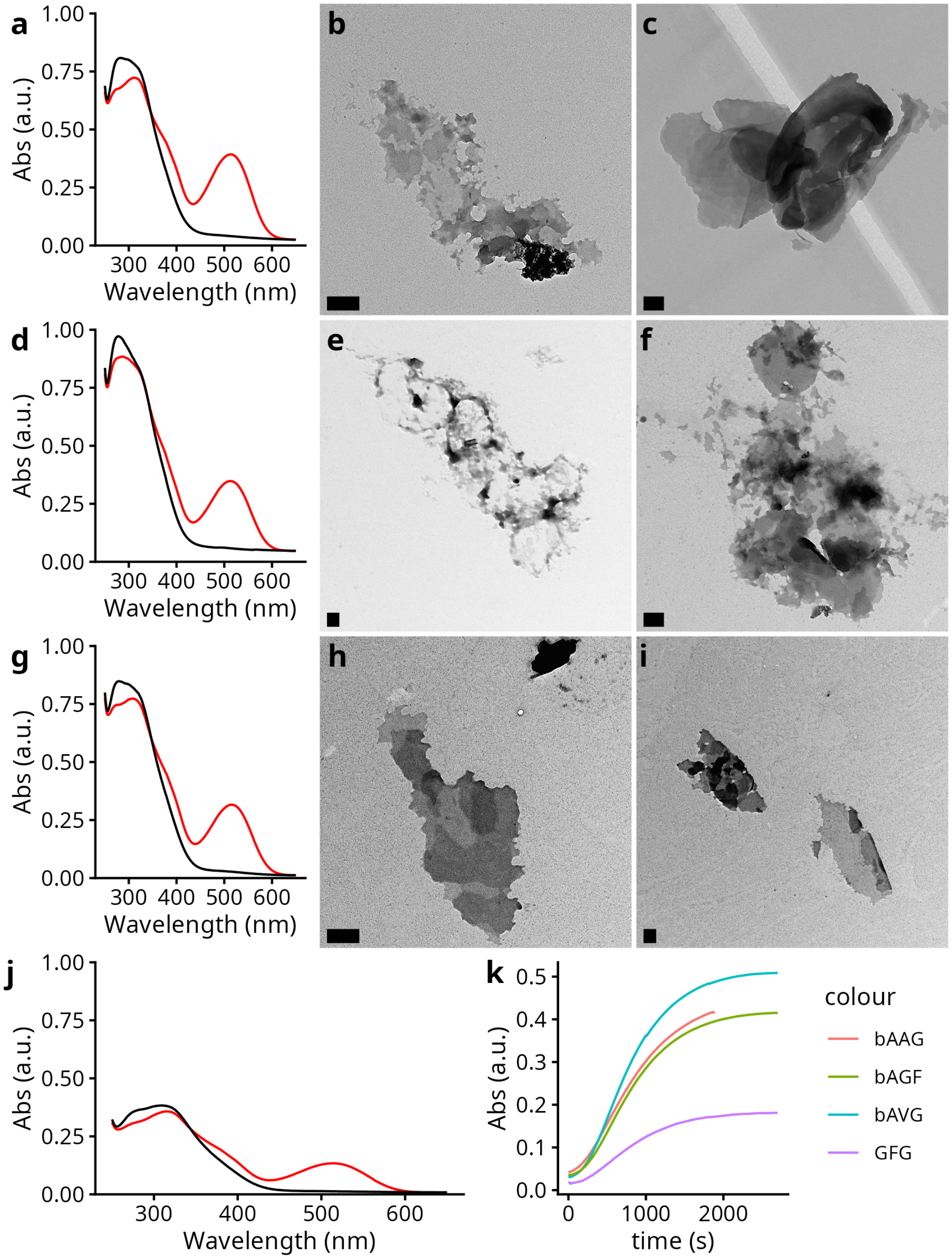

Figure S4: UV spectra before (red) and after (black) irradiation for a) βAla-Ala-Gly, d) βAla-Val-Gly, g) βAla-Gly-Phe and j) Gly-Phe-Gly; TEM micrographs before irradiation for b) βAla-Ala-Gly, e) βAla-Val-Gly, h) βAla-Gly-Phe; TEM micrographs after iradiation for c) βAla-Ala-Gly, f) βAla-Val-Gly, i) βAla-Gly-Phe. Samples were prepared in MES 20 mM pH 6 at a concetration of 120 ţM (βAla-Ala-Gly, βAla-Val-Gly, βAla-Gly-Phe) and 60 µM (Gly-Phe-Gly). Scale bars denote 200 nm; k) Thermal isomerization at 45 °C.

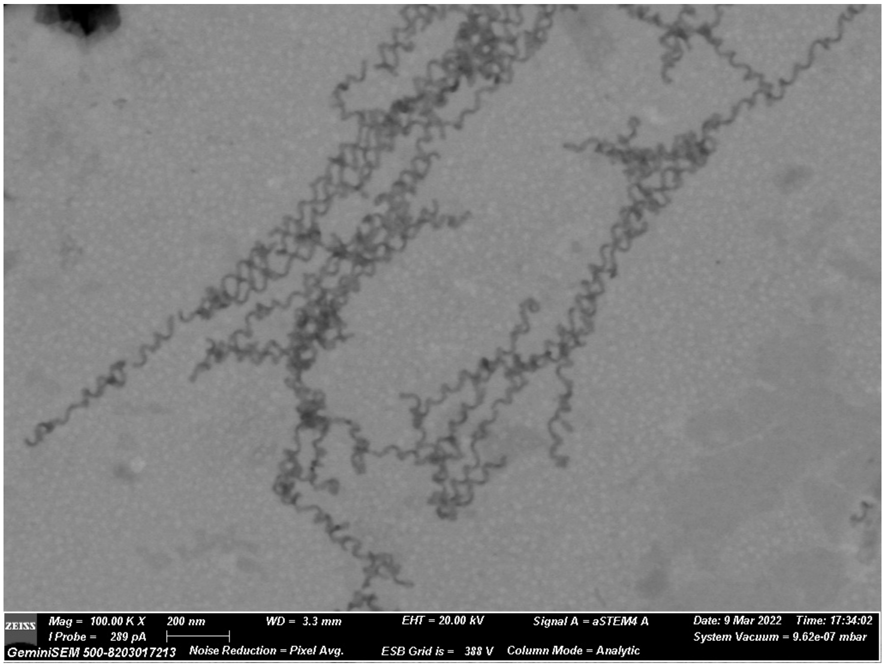

Figure S5: Helical nanorribbons formed by sequence SPβAla-Phe-Glu

S2.2 Light Triggered Isomerization

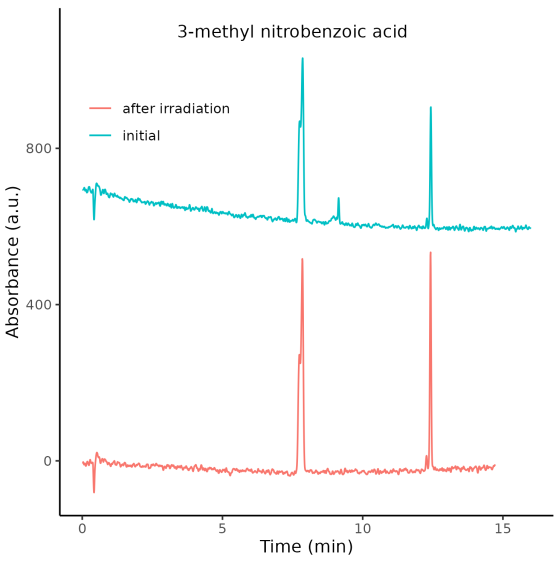

Figure S6: uHPLC chromatograms in the presence of 3-methyl nitrobenzoic acid as internal reference before and after irradiation. Gradient (H_2O-0.1%TFA-acetonitrile-0.1% TFA 95:5 (0 min) to 5:95 (15 min)).

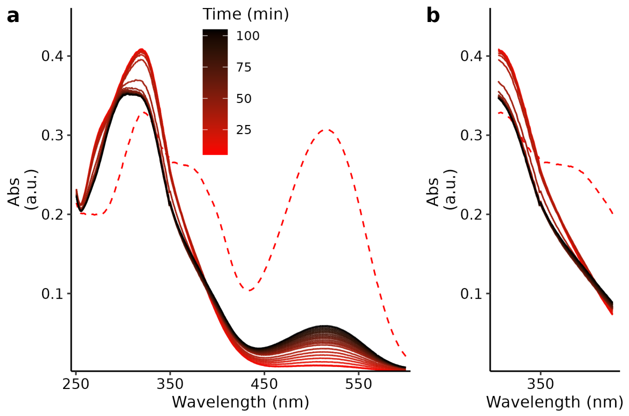

Figure S7: Evolution of UV/vis absorption spectra after SP conversion (110 µM). Dashed lines denote the UV/vis spectra acquired before irradiation; a) complete spectra; b) magnification at lower wavelengths were an initial isosbestic point is lost by concomitant self-assemby.

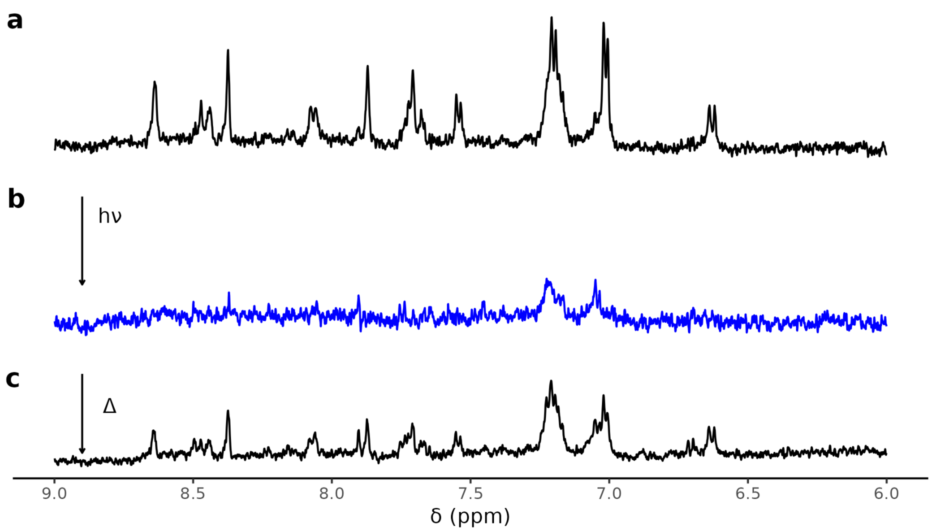

Figure S8: ^{1}H NMR (500 MHz, D_2O, 298 K, water suppresion) of peptide 1 mM in MES 50 mM pH 6 a) before, b) after white light irradiation and c) after 30 min equilibration in the dark at 45 °C.

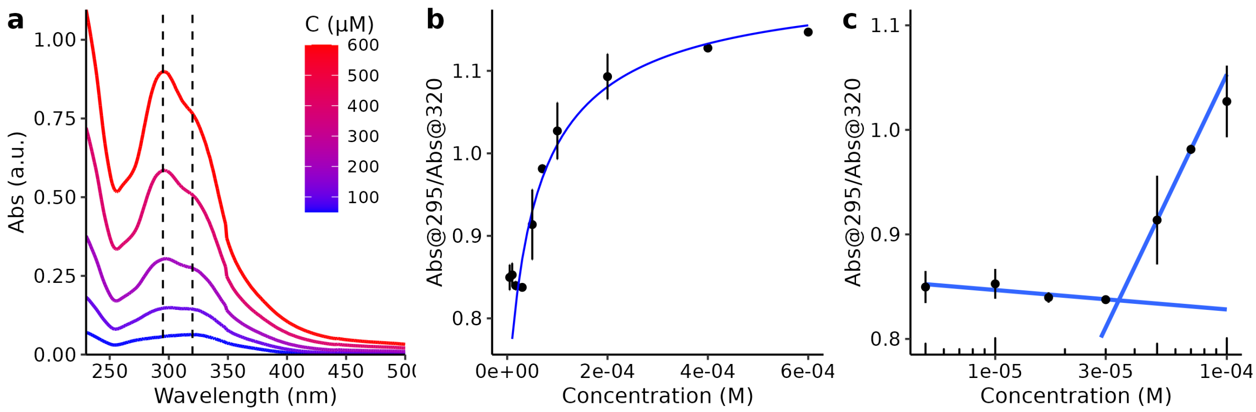

Figure S9: Estimation of the polymerization by UV-vis spectroscopy. a) UV-vis spectra of pre-assembled (SP form) **SP** at increasing concentration (MES 20 mM, pH 6) 25 °C. All samples were irradiated with broadband white light (38 cd) at room temperature until complete decoloration (> 1 min). A shift of the absorption maximum is observed upon increasing the concentration. At lower concentration the maximum is locate at *ca* 320 nm, while at higher concentrations the maximum is located at *ca* 295 nm (dashed lines); b) Ratio of the absorbance at 320 and 295 nm *versus* concentration; c) Magnification of the relative absorbance *versus* concentration showing a threshold in the spectroscopic features. Blue lines denote fitting to a linear equation.

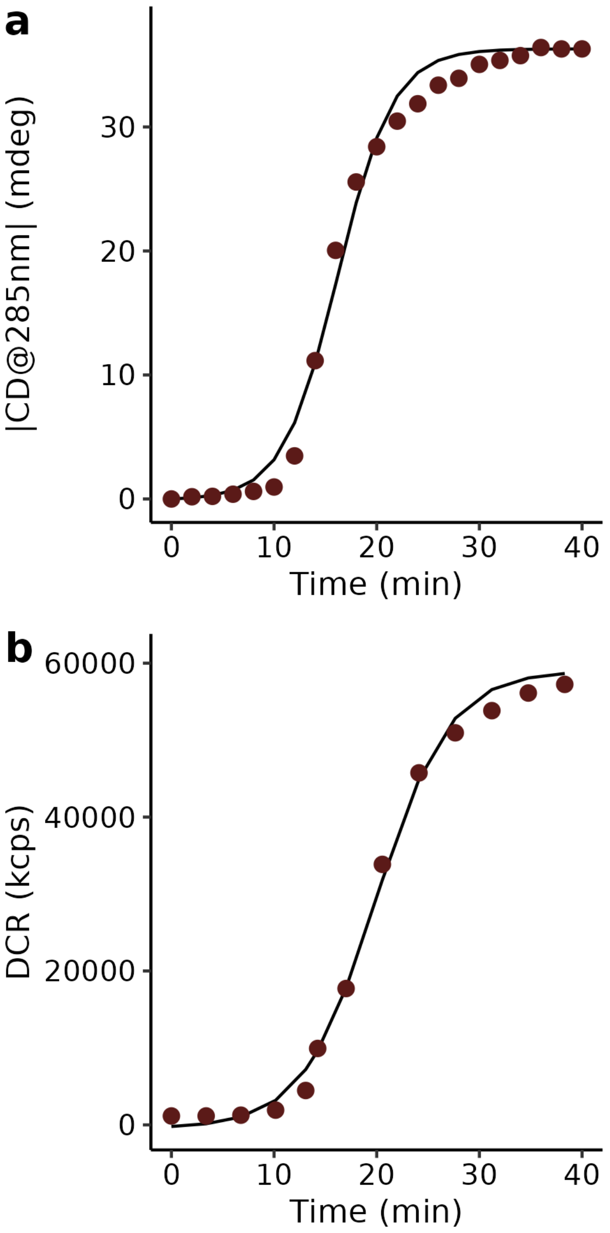

Figure S10: Assembly of **SP** 180 μM in MES (20 mM, pH 6) monitored by a) CD and b) DLS at a temperature of 20 °C after a irradiation pulse (10-15 s, broadband white light, 38 cd). Dots denote experimental points and lines correspond to the fitting to a simple autocatalytic model (see Section S1.3, determination of the autocatalyitic kinetic constants).

S2.3 Thermal Relaxation of the Photogenerated Spiropyran State

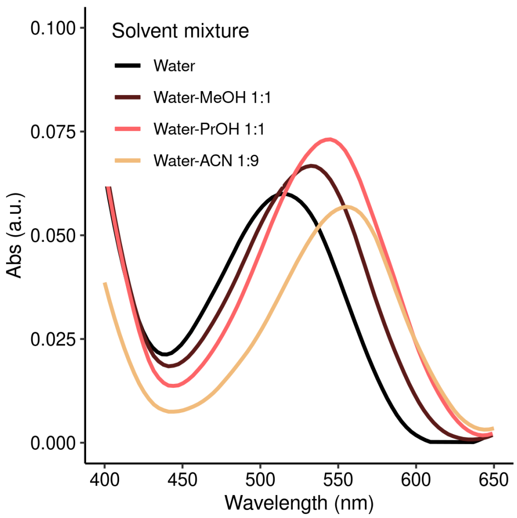

Figure S11: UV-visible spectra of the merocyanine region for **MCH-SP** (80 µM in MES 20 mM pH 6) in different solvent mixtures, including protic (MeOH, PrOH) and aprotic (ACN). In all cases, the presence of organic solvents produces a bathochromic shift of the merocyanine maximum absorption wavelength. Path length is 0.2 cm.

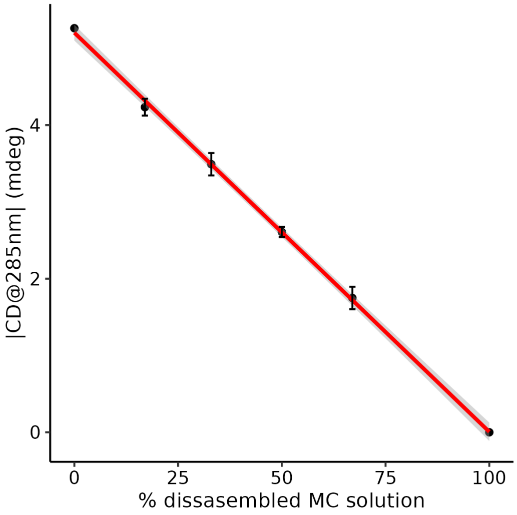

Figure S12: Extent of coassembly of the spiropyran and merocyanine peptide forms determined by circular dichroism at room temperature. The total concentration of **SP** is constant (870 µM in MES 20 mM pH 6) while the volume ratio of the non-irradiated (**MCH**) and assembled (**SP**) sample were mixed at different ratios. Here "%disassembled MCH solution" denotes the percentaje the MC solution obtained after preparing the peptide solution and before irradiation (see Figure 2b in the manuscript). CD was monitored until signal stabilization. The red line denotes linear fitting to the experimental points, and the grey shading corresponds to the 95% confidence interval.

Transient self-assembly of SP and thermal isomerization Observed kinetic constants corresponding the thermal isomerization from the spiropyran towards merocyanine isomer were obtained from UV-visible kinetic experiments, as described before (see reference 40 from manuscript):

$$A_t = A_\infty - [A_\infty - A_0] \times \exp(-k_{obsd} t) \qquad \text{(S9)}$$

where A_t is the absorbance of the merocyanine isomer at time t, A_∞ is the absorbance when $t \to \infty$, A_0 is the absorbance at $t = 0$ and k_{obsd} is the observed kinetic constant which contains the sum of the forward and reverse isomerization constants. Fitting to data is shown in Figure S13, and the calculated constants are shown in Table S4.

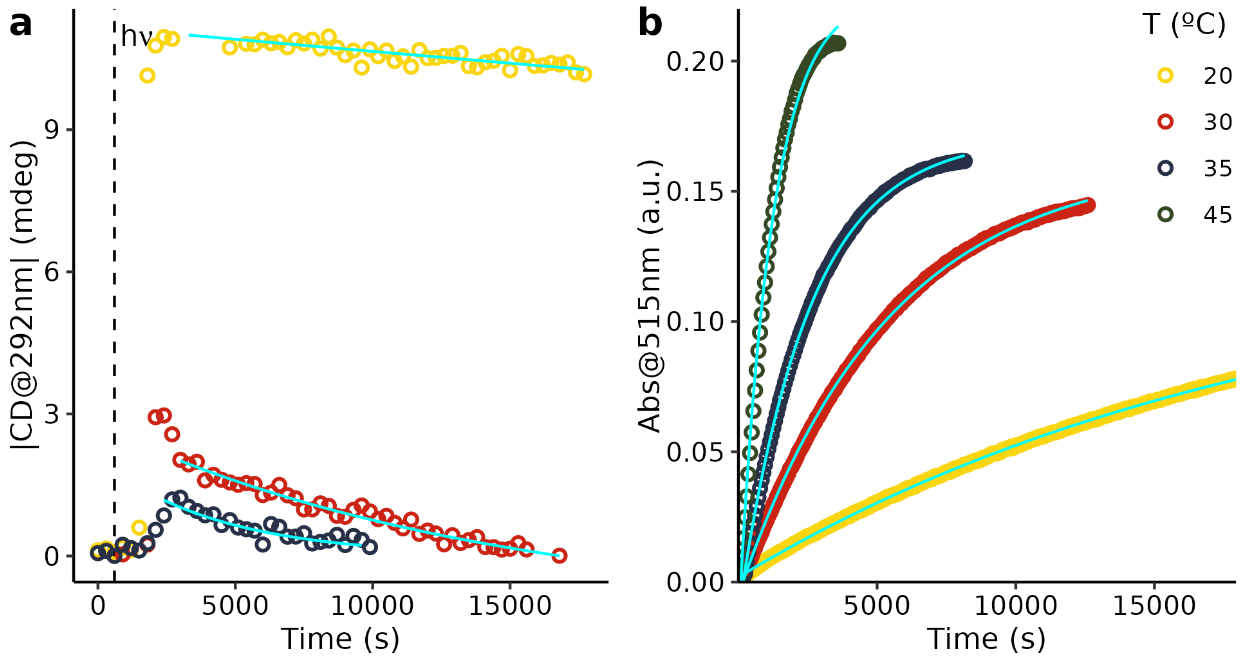

Figure S13: a) Transient assembly of **SP** (110 µM in MES 20 mM, pH 6) monitored by CD. Samples containing were briefly irradiated with broadband white light (10-15 s, 38 cd, dashed line) and allowed to evolve at different temperatures. A wavelength of 292 nm was chosen in order to capture spectral changes in the excitonic coupling signals. Temperature is denoted as colors shown in the legend (right panel); b) Thermal isomerization of the spiropyran isomer of **SP** (110 µM in MES 20 mM, pH 6) monitored by UV spectroscopy at different temperatures. The formation of the merocyanine isomer was monitored through the absorbance increase of the band located at 515 nm. Circles denote experimental points, and lines indicate fitting to a first order reaction kinetic model described above. The path length was 0.2 cm.

T (°C)	$t_{1/2}$ (min)
20	2442
30	166
35	57

Table S3: Half-life time calculated from the fitting of the decay of the CD signal to a monoexponential kinetic model ($CD = CD_{init} \times e^{-kt}$), where CD_{init} is the CD signal a the start of the decay, k is the kinetic rate decay constant and t time.

T (°C)	k_{obsd} (1/s)	A_∞ (a.u.)	$t_{1/2}$ (min)
20	$(5.12 \pm 0.04) \times 10^{-5}$	0.1280 ± 0.0001	225
30	$(1.88 \pm 0.01) \times 10^{-4}$	0.1620 ± 0.0004	62
35	$(3.98 \pm 0.03) \times 10^{-4}$	0.1710 ± 0.0004	29
45	$(8.4 \pm 0.2) \times 10^{-4}$	0.225 ± 0.0002	14

Table S4: Calculated observed kinetic constants and maximum absorbance for thermal isomerization of the spiropyran isomer of **SP**. See conditions in Figure S13

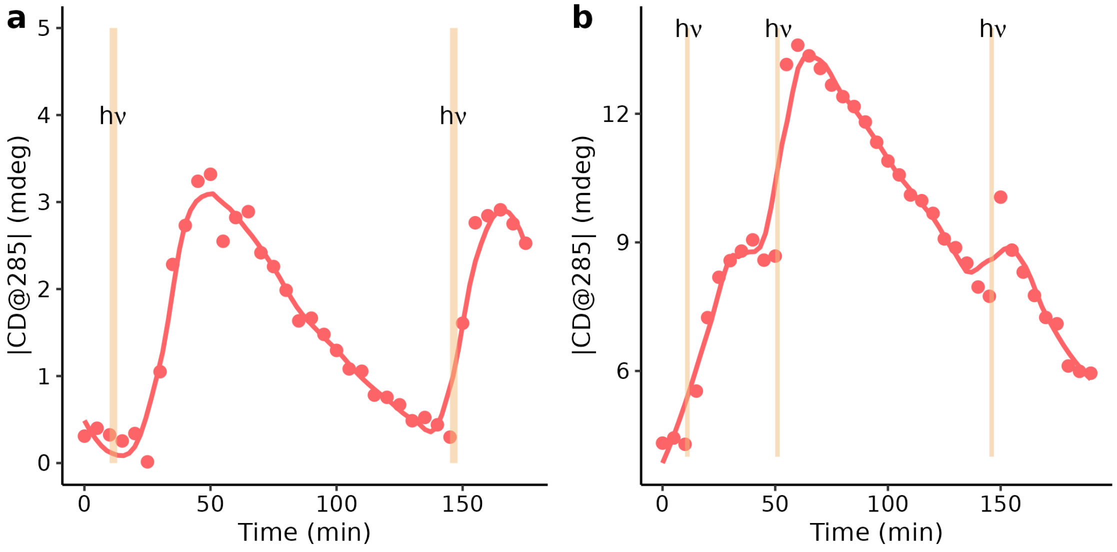

Figure S14: Dynamic assembly of SP after different irradiation sequences (110 μM **SP** in MES 20 mM, pH 6, 30 °C). Irradiation were carried out with a broadband white light (38 cd); the irradiation time is indicated by the filled areas: a) Reversible CD signal after irradiation and relaxation. Smooth lines are for eye guidance only; b) Sequential assembly after short pulses that stepwise increase the concentration of **SP**.

S2.4 Self-Assembly of the Peptide Photoswitch

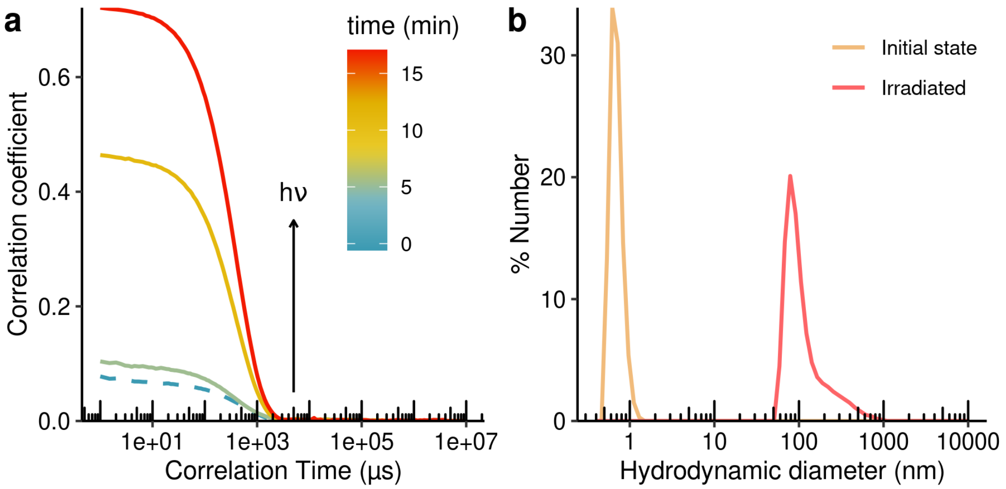

Figure S15: Assembly of **SP** 110 µM in MES (20 mM, pH 6) monitored by DLS at a temperature of 20 °C after an irradiation with broadband white light (10-15 s, until complete decoloration, 38 cd): a) Correlation function. Dashed blue line indicates the correlation function before irradiation; b) Size distribution before and after irradiation expressed as number percentage.

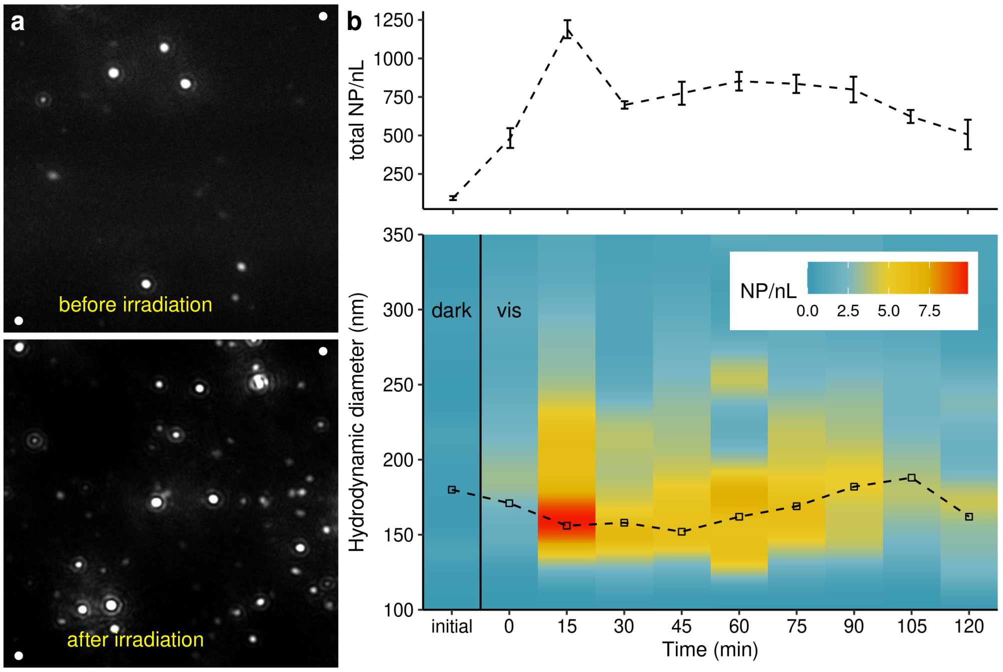

Figure S16: Assembly of **SP** 130 µM in MES (20 mM, pH 6) monitored by NTA at a temperature of 20 °C. The vis region denote a irradiation with broadband white light (10-15 s, until complete decoloration, 38 cd): a) Representative images of the nanoassembly dispersion before (top) and after (bottom) irradiation; b) Evolution of the total number of particles and size distribution after irradiation.

Self-assembly monitored by STEM and cryoTEM microscopies Solutions prepared to containg high concentration of **MCH** where visualized by STEM and cryoTEM microscopies before and after irradiation. Before irradiation (Figure S17a,b and Figure S18a), only amorphous aggregates (cryoTEM) and staining residues (STEM) were observed. After irradiation (Figure S17c,d and Figure S18b), clustered tape-like nanostructures are generated, with a coverage of $2.3 \pm 0.9\%$ evidenced in STEM images. After equilibration in the dark, a decrease in the population of tape-like aggregates is observed, as well as the emergence of amorphous aggregates (Figure S18c).

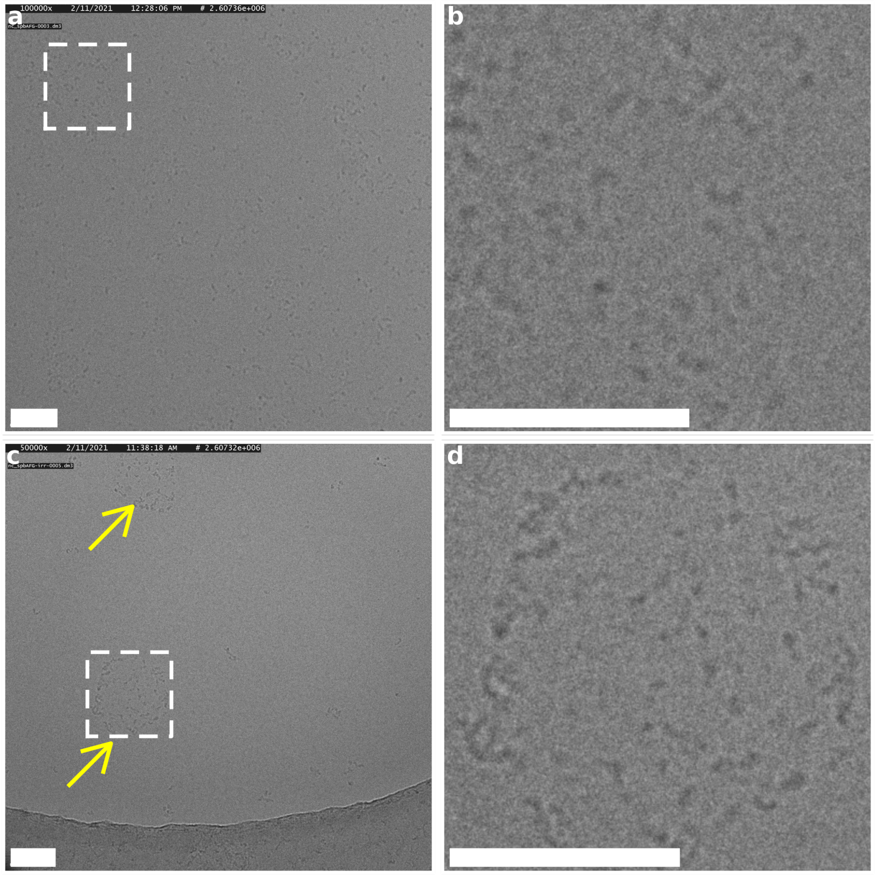

Figure S17: CryoTEM micrographs showing light-triggered self-assembly of **SP** (MES 20 mM, pH 6): a) before and c) after irradiation with visible light. Dashed boxes indicate magnified regions b) before and d) after irradiation. Arrows indicate local clustering of aggregates. Similar tape-like structures are observed at higher concentrations and temperature (500 μM **SP**, 45 °C), particularly at short irradiation times (30 min, Figure 3a). Scale bars: a), b) 50 nm; c), d) 100 nm.

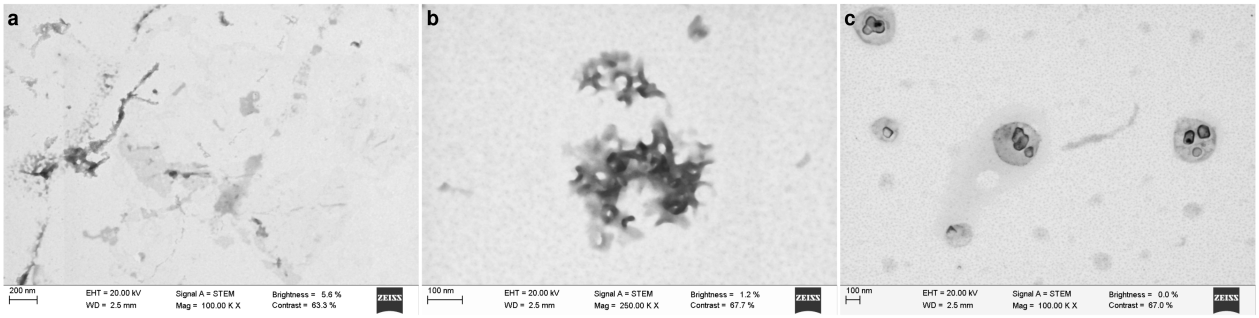

Figure S18: STEM micrographs for the assembly of **SP** (140 µM in MES 20 mM, pH 6, 20 °C) a) before light visible irradiation; b) after irradiation with a broadband white light (10-15 s, until complete decoloration, 38 cd) and equilibration of the CD signal, c) after overnight equilibration at dark at room temperature.

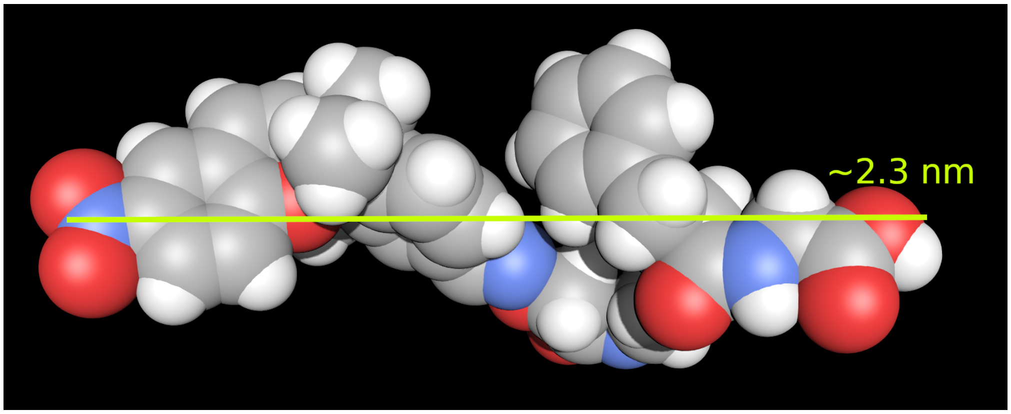

Figure S19: CPK model of **SP** after geometrical optimization using a MMFF94s force field and approximate molecular length.

S2.5 Formation of out-of-equilibrium Chiral Polymorphs by One Irradiation Cycle

Heating and evaporation controls

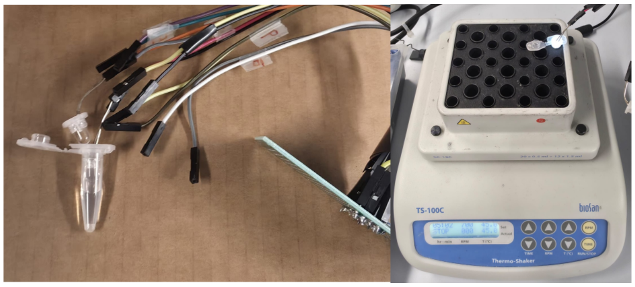

Time (min)	Temperature (°C)
5	43.9 °C
15	43.9 °C
30	43.9 °C
60	44.1 °C
120	43.8 °C
180	43.7 °C

Table S5: Sample heating due to LED irradiation. The image shows the experimental setup used.

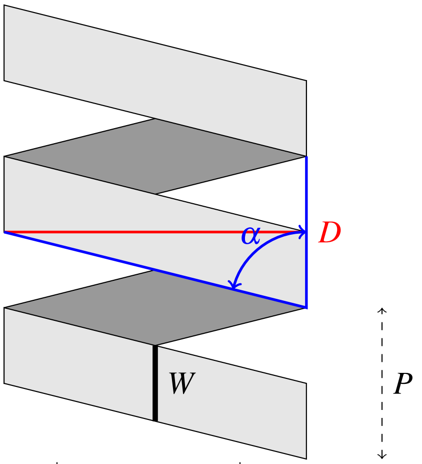

Tape Width W (nm)	Angle α (°)	Pitch P (nm)	Diameter (nm)
29 ± 8	59 ± 7	72 ± 15	57 ± 8

Table S6: Summary of geometrical feature ranges of helical nanoribbons determined by TEM micrographs analysis.

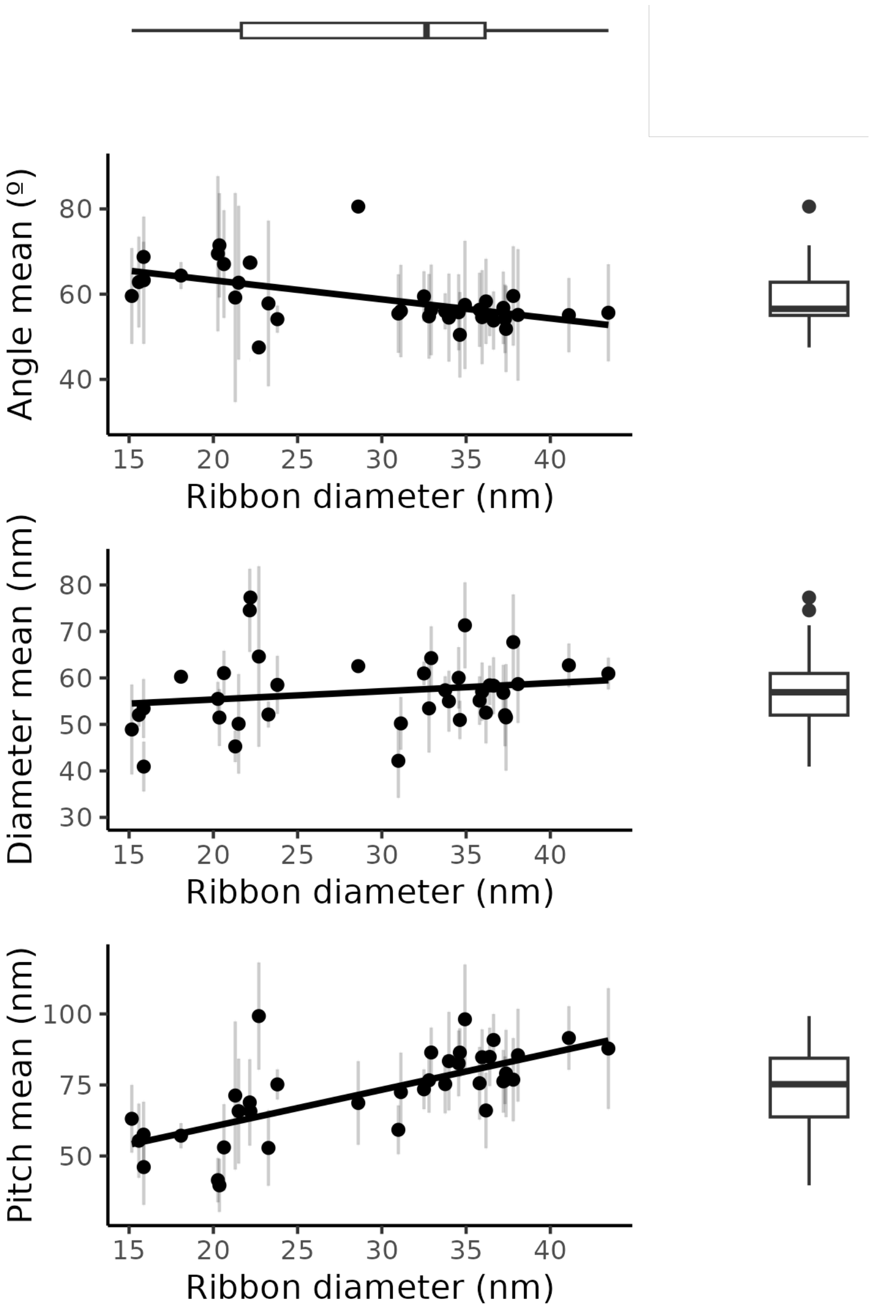

Figure S20: Correlation of geometrical features in helical nanoribbons determined by TEM micrographs analysis. **SP** (500 μM, MES 50 mM pH 6) was assembled by continuous irradiation with broadband visible light (10 cd) for 3 h at 45 °C. Regression coefficients for angle *vs* width and pitch *vs* width are significantly differ from 0, while diameter *vs* width does not.

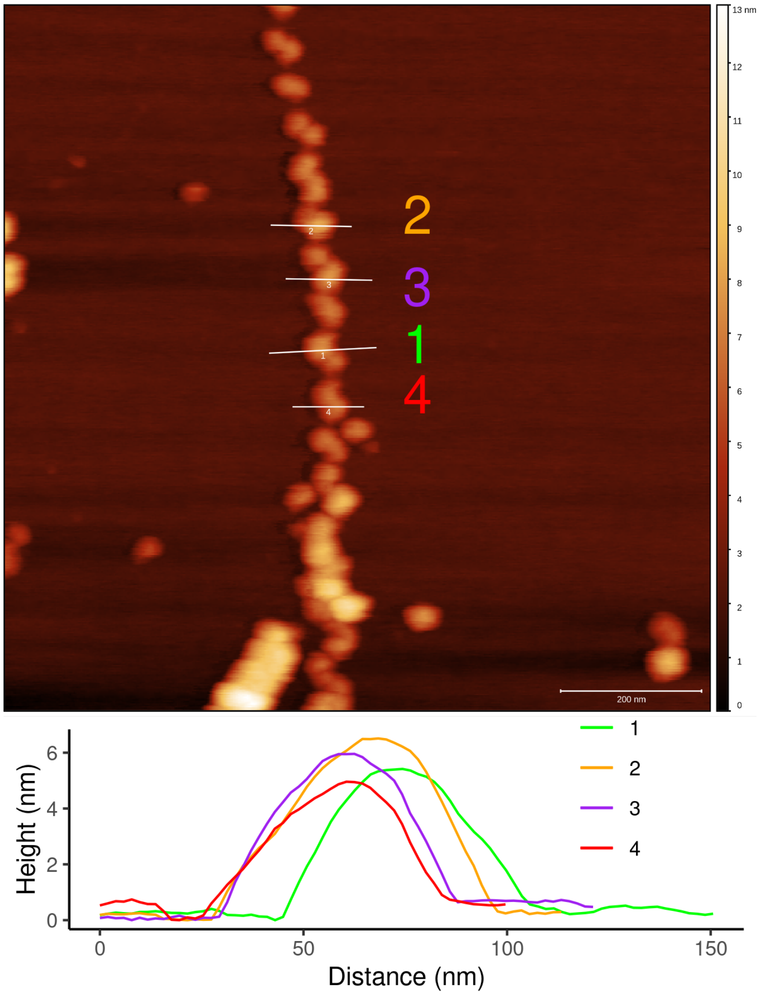

Figure S21: AFM micrographs of **SP** (500 μM, MES 50 mM, pH 6) assembled by continuous irradiation with broadband white light (10 cd) at 45 °C for 3 h.

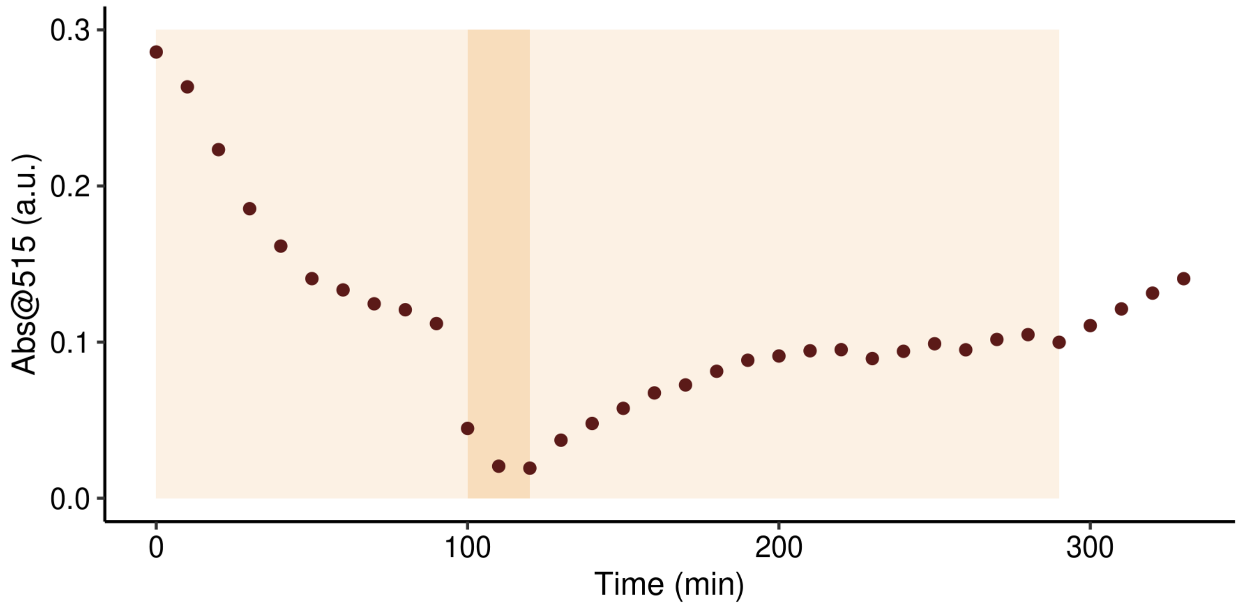

Figure S22: Evolution of **MCH** upon PWM changes using a broadband white light (10 cd), monitored by UV. Sample was kept at 30 °C (125 µM in MES 20 mM pH 6). Faintly shaded and darker shaded areas correspond to 4% and 60% duty cycles respectively. No shading indicates no irradiation.

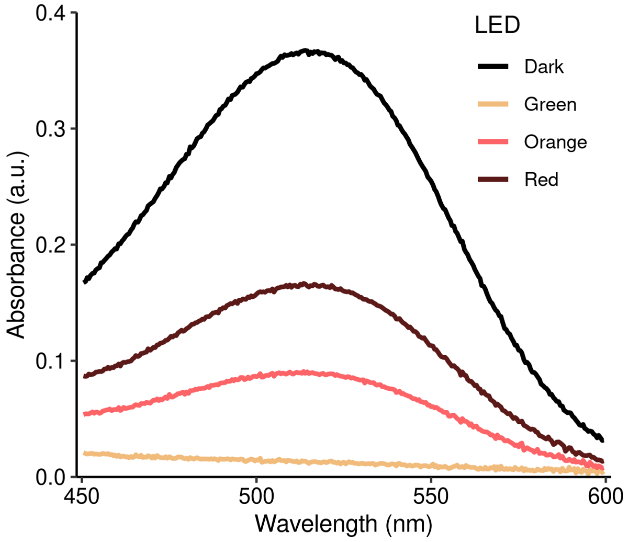

Figure S23: UV spectra at the merocyanine region of **SP** (0.5 mM, MES 50 mM pH 6) after equilibration (2 h) at 45 °C under continuous irradiation with different LEDs and duty cycles (100 %, 20% and 20 % for green (λ_{max} = 525 nm, 20 cd), orange (λ_{max} = 590 nm, 7 cd) and red (λ_{max} = 629 nm, 10 cd) LEDs respectively).

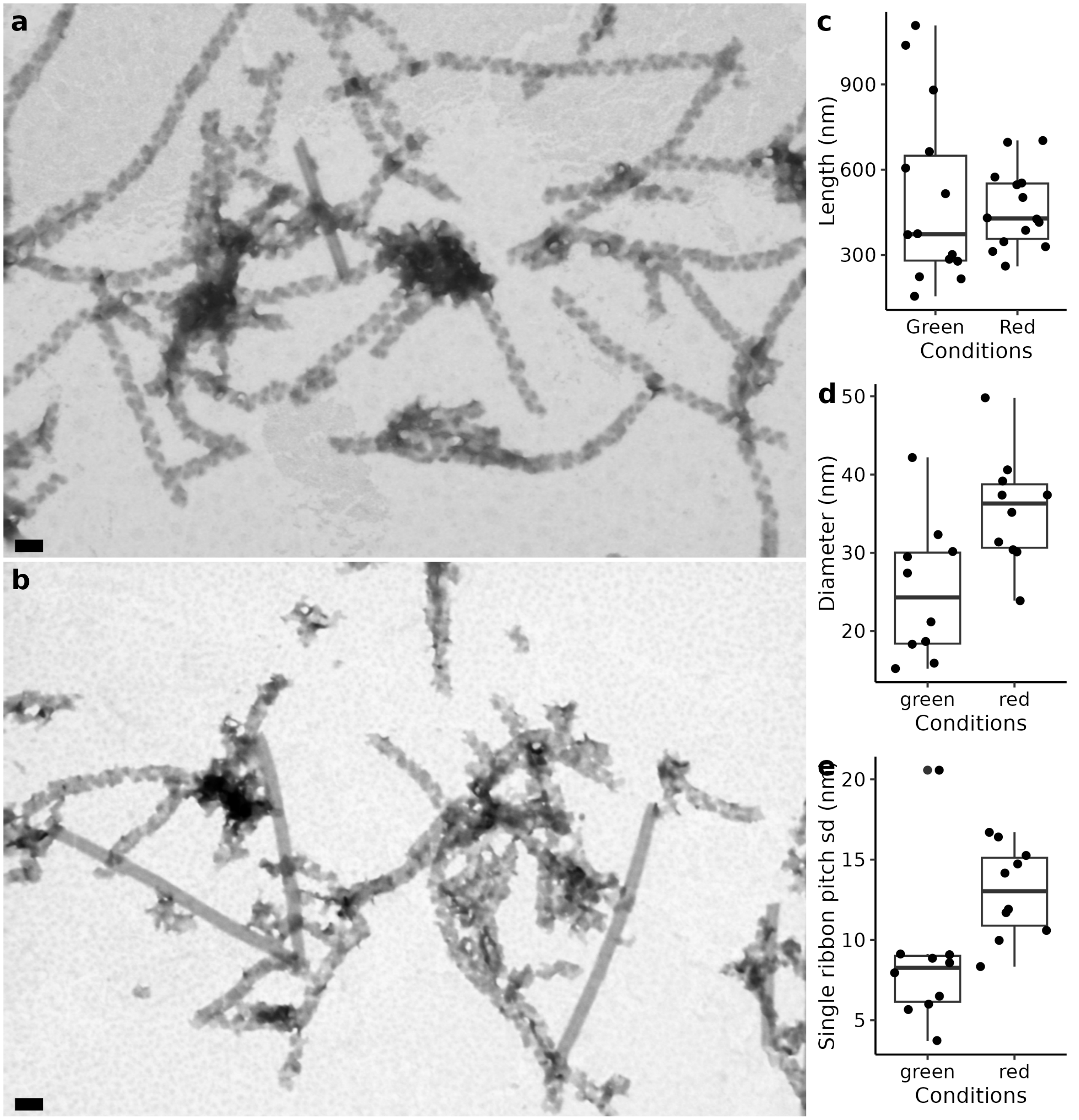

Figure S24: STEM micrographs for samples continuously irradiated with different commercial LED wavelengths and duty cycles: a) Irradiated with green (λ_{max} = 525 nm, 20 cd) LEDs and 100 % duty cycle; b) Irradiated with red (λ_{max} = 629 nm, 10 cd) LEDs and 20 % duty cycle. In all cases, the concentration of **SP** was 0.5 mM (MES 50 mM, pH 6) and samples were assembled by continuous irradiation of 3 h at 45 °C; c) Length distribution; d) Width distribution; e) single nanoribbon pitch standard deviation, i.e. the standard deviation calculated from all the pitches measured for a single nanoribbon.

S2.6 Enhancement of Structural Evolution by Cycles of Irradiation and Dark Relaxation

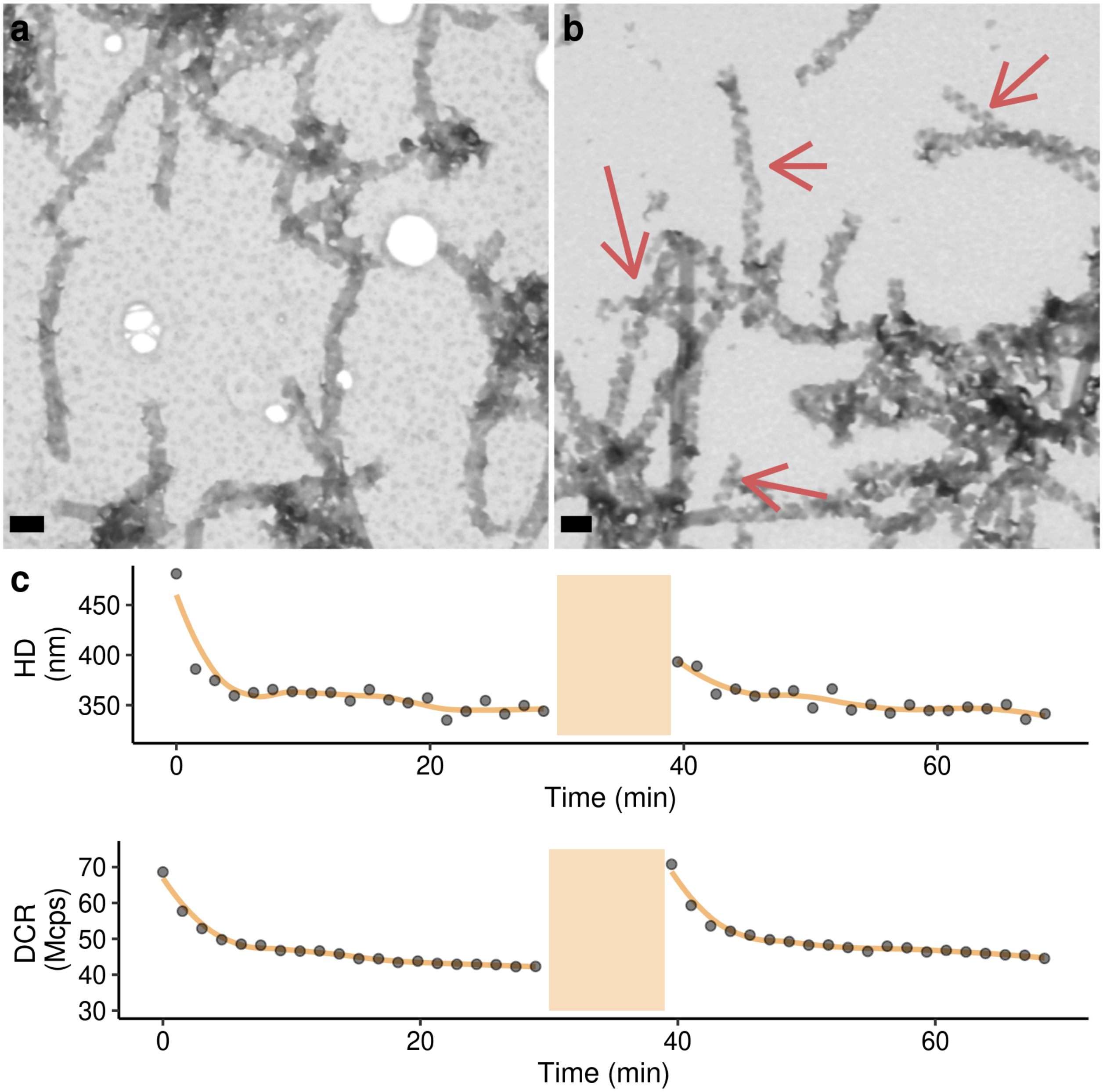

Figure S25: Recovery of helical nanoribbons after irradiation with visible light: a) A solution of **SP** was assembled under continuous broadband white light irradiation (10 cd) for 3 h at 45 °C (0.5 mM, MES 50 mM, pH 6), then allowed to relax at dark at the same temperature for 1 h.; b) this solution was irradiated continuously with broadband visible white light for 3 h.; c) Time-dependent derived count rate upon thermal relaxation (45 °C). The yellow box indicates re-irradiation for the corresponding sample at the same temperature.

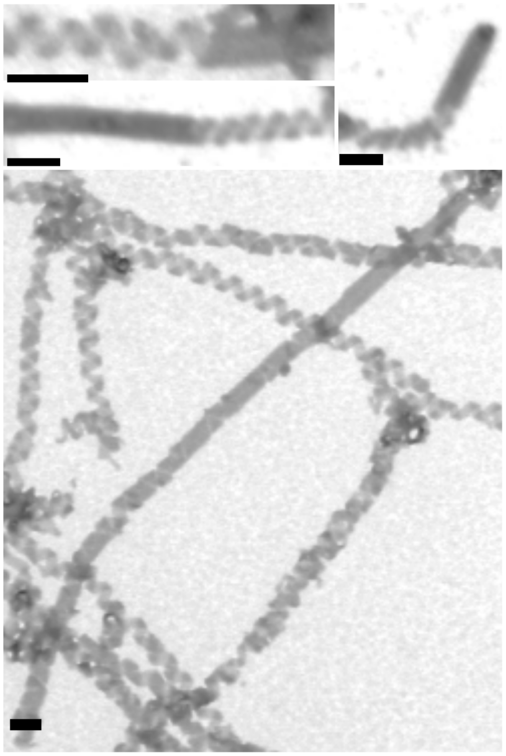

Figure S26: Examples of nanotube-helical nanoribbon transitions undergoing on single ribbons. Scale bars are 100 nm.

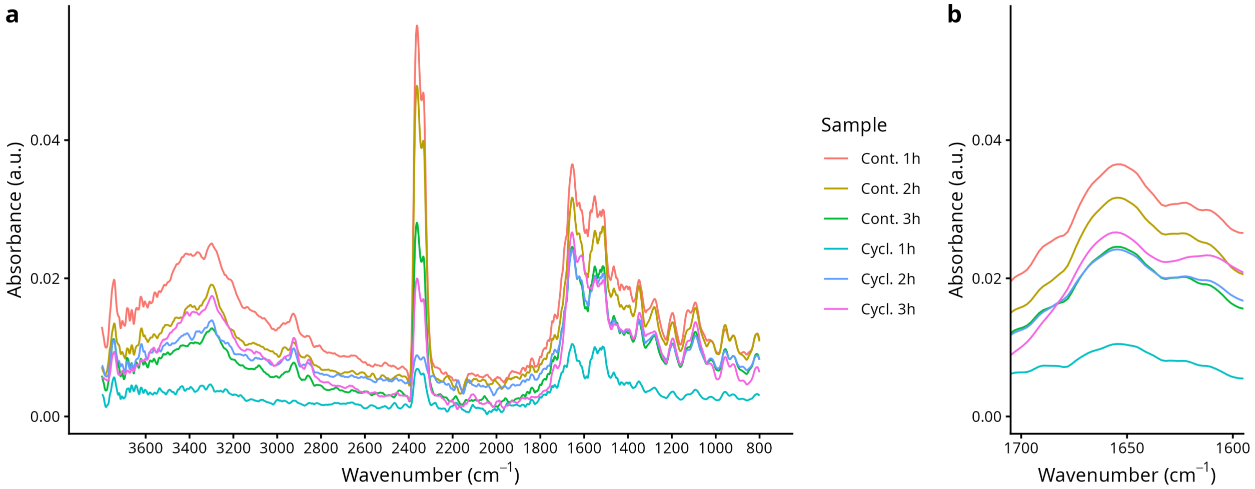

Figure S27: a) FTIR spectra of peptide assembled by continuous irradiation and over light-dark cycles; b) magnification of the amide I region

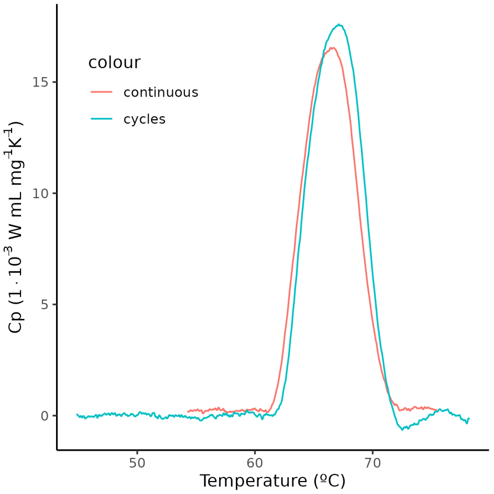

Figure S28: DSC measurmenets of peptide after continuous irradiation or after light-dark cycles.

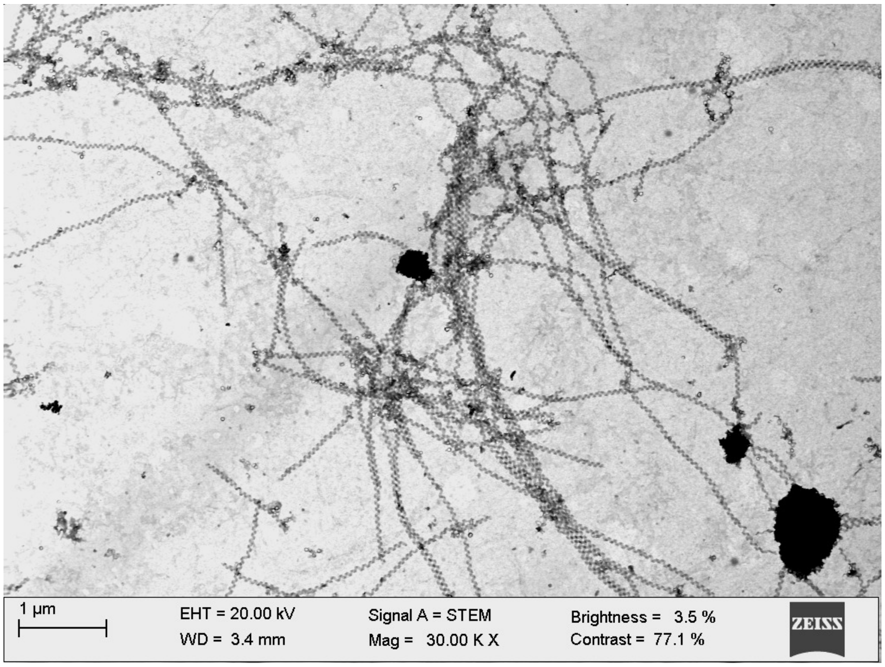

Figure S29: Helical nanoribbons formed from **SP** (500 µM, MES 50 mM, pH 6) using 15 min white light-dark cycles (broadband white light, 10 cd)

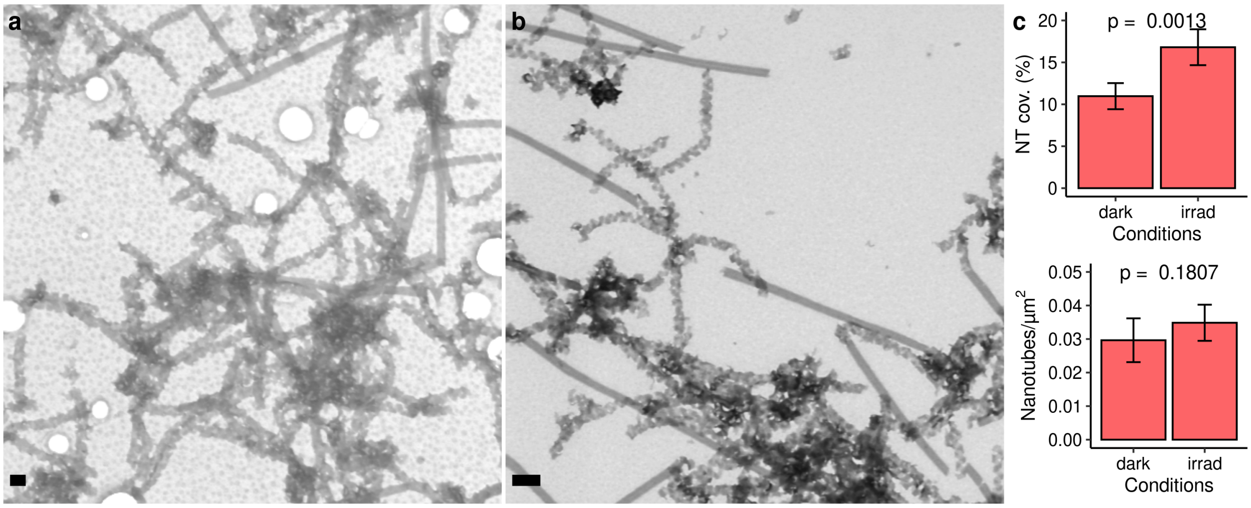

Figure S30: Conversion of nanotubes to nanoribbons from a nanoribbon solution equilibrated at dark (500 μM in MES 50 mM, pH 6): a) Initial state; b) after continuous irradiation with broadband white light (10 cd) for 3 h; c) quantification of nanotube coverage and nanotube density. In both cases, no indication of nanotube conversion is observed. Scale bars denote 100 nm.

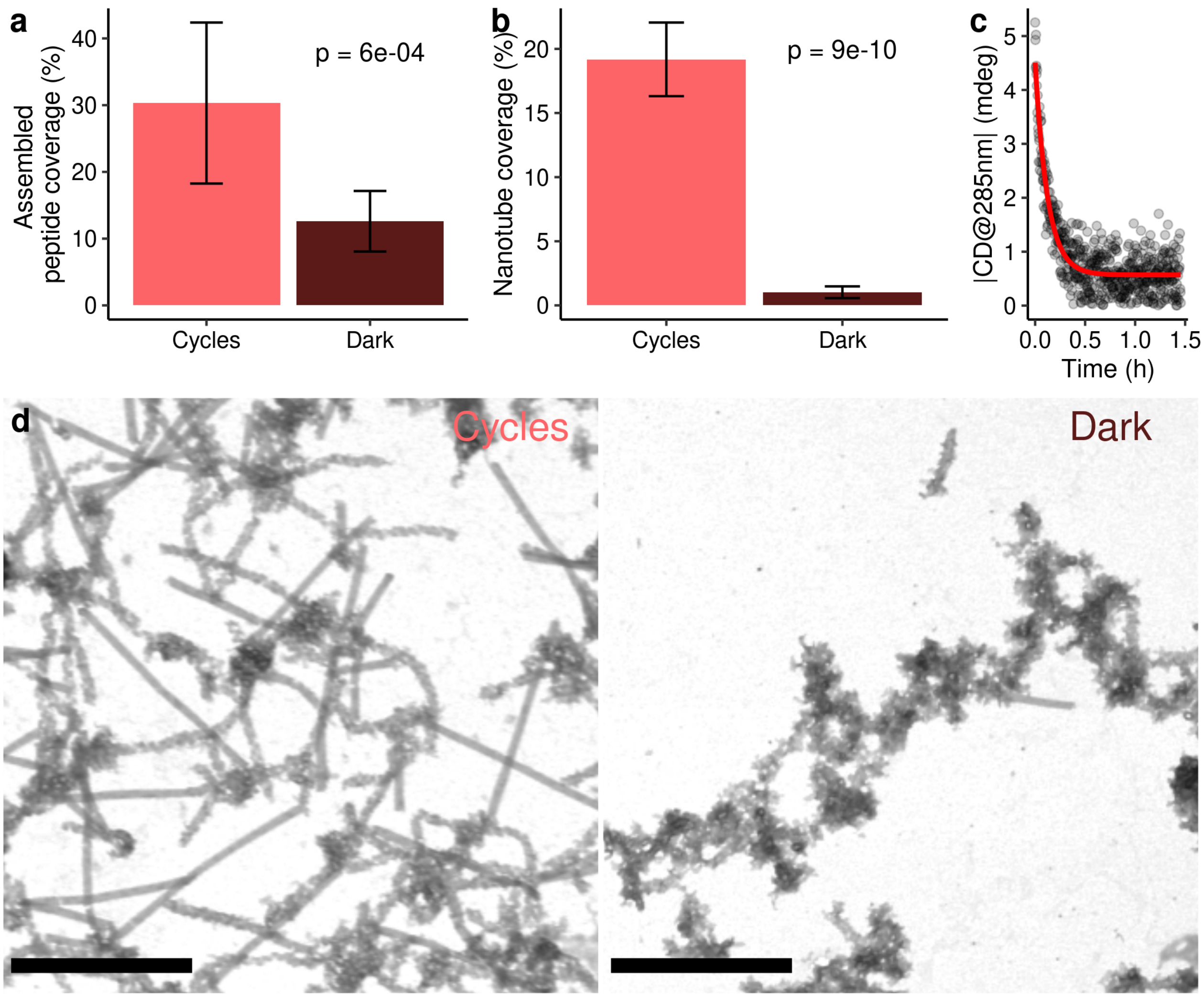

Figure S31: Structure disassembly probed by microscopy and spectroscopy: a) Decrease of assembled peptide coverage (0.5 mM solution in MES 50 mM pH 6) after equilibration at 2 h in the dark at 45 °C of a sample subjected to light-dark cycles; b) Decrease of nanotube coverage in the same conditions ; c) disassembly at 45 °C in the dark for a preassembled sample (150 μM in MES 25 mM pH 6). The red line is the fitting to a exponential decay model; d) Micrograph examples for the data collected in a) and b). Averages were collected from 11 and 15 micrographs of 65.3 μm^2 area. Scale bar denote 1 μm

S3 Characterization of new derivatives

S3.1 1

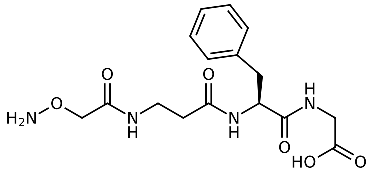

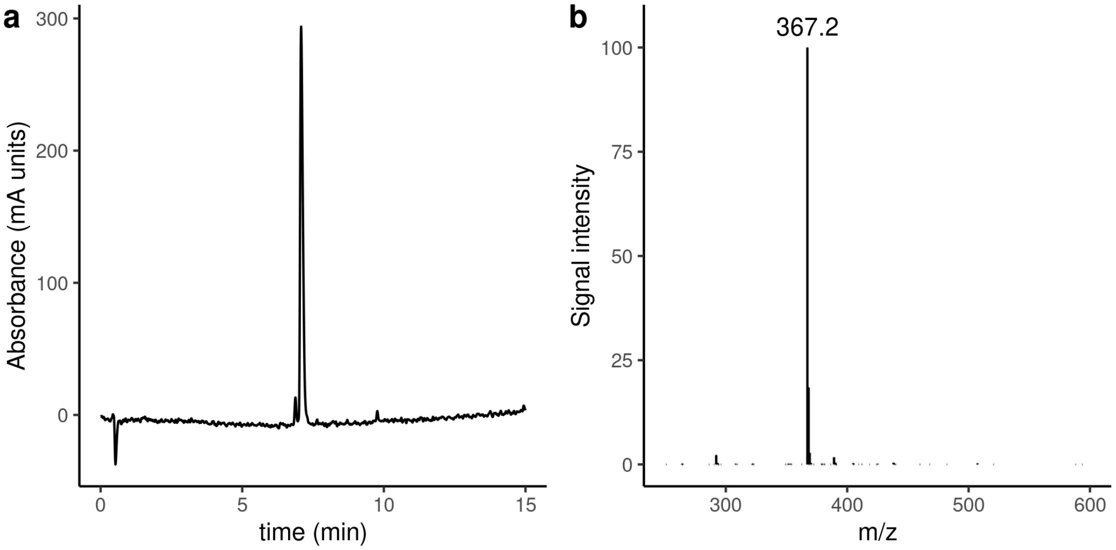

Figure S32: a) Chromatogram of derivative **1** (H_2O-0.1% TFA-acetonitrile-0.1% TFA 95:5 (0 min) to 5:95 (15 min)); b) Mass spectra of the more intense band. Predicted mass for $C_{16}H_{23}N_4O_6^+$: 367.16 amu

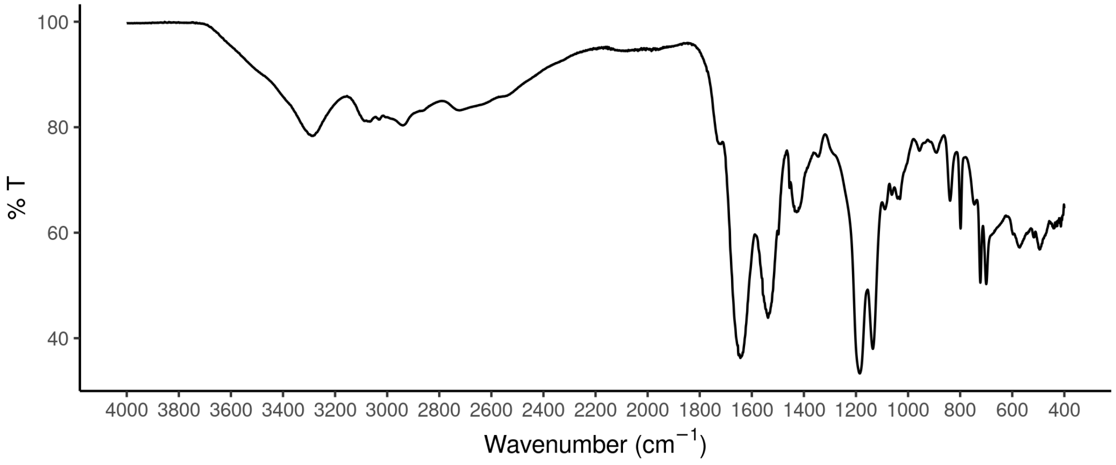

Figure S33: IR-ATR spectra of solid **1**

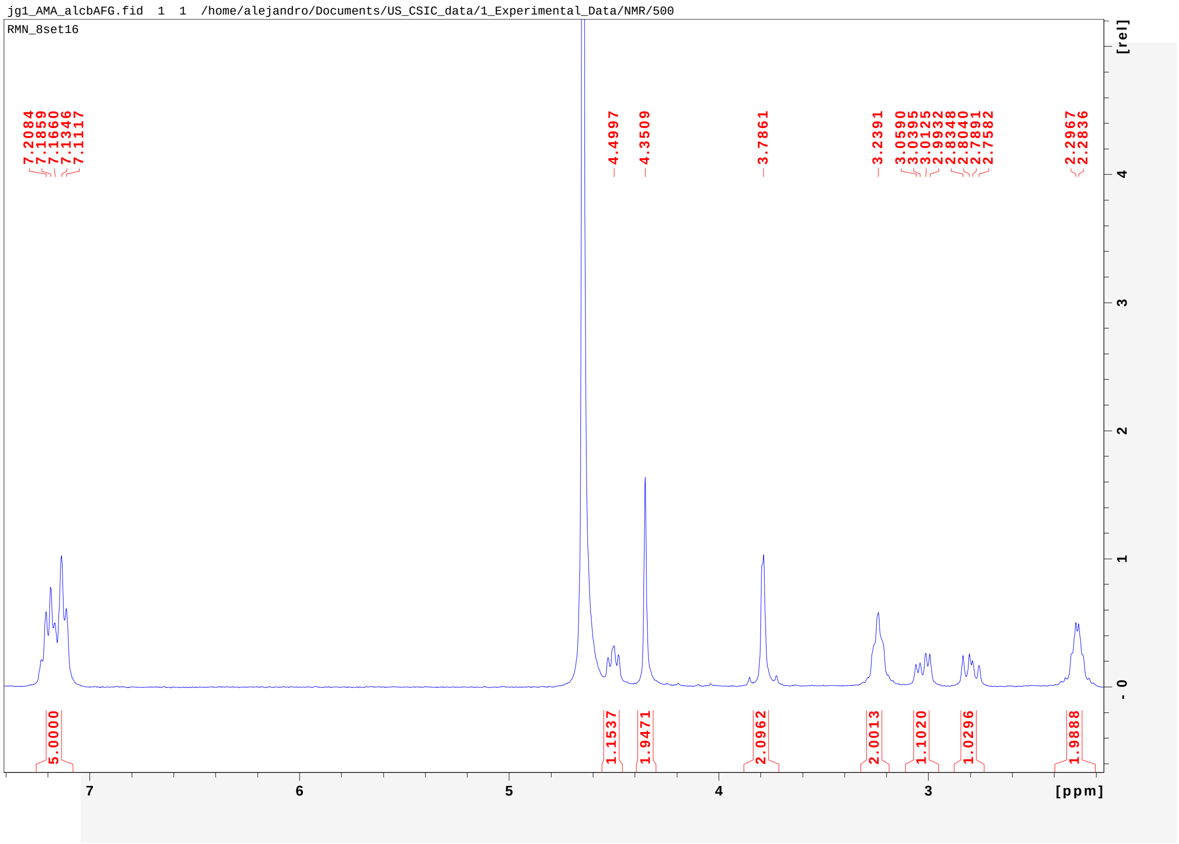

Figure S34: ^{1}H NMR (300 MHz, D_2O 1:1, 293 K) of derivative **1**

S3.2 SP

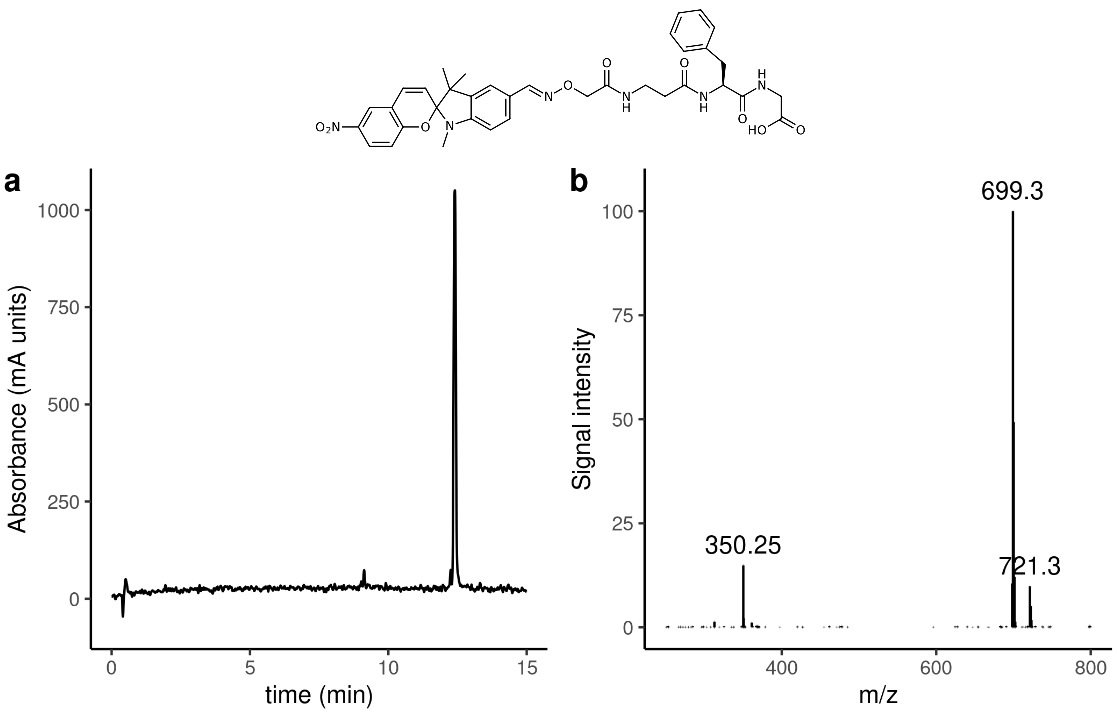

Figure S35: Chromatogram of derivative **SP** (H_2O-0.1% TFA-acetonitrile-0.1% TFA 95:5 (0 min) to 5:95 (15 min)); b) Mass spectra of the more intense chromatographic band. Predicted mass for $C_{36}H_{39}N_6O_9^+$: 699.28 amu

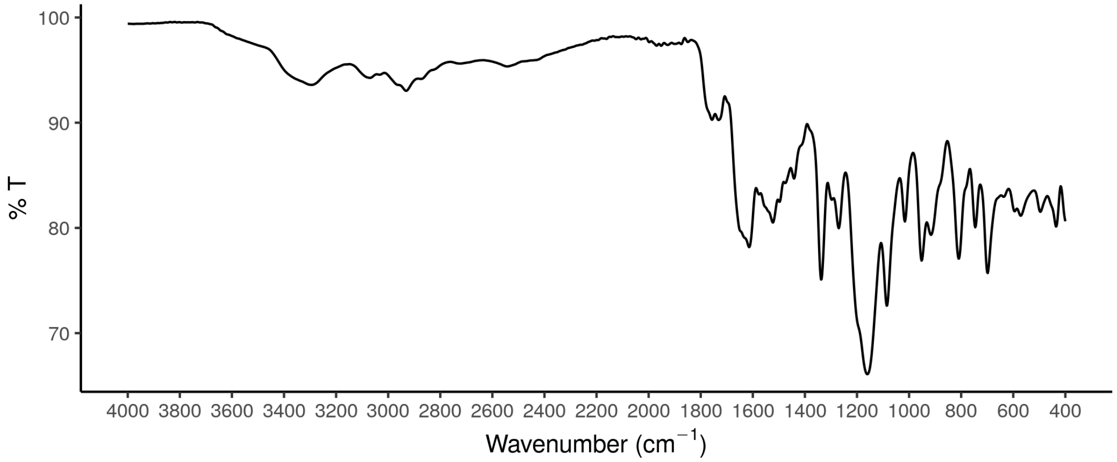

Figure S36: IR-ATR spectra of solid **SP**

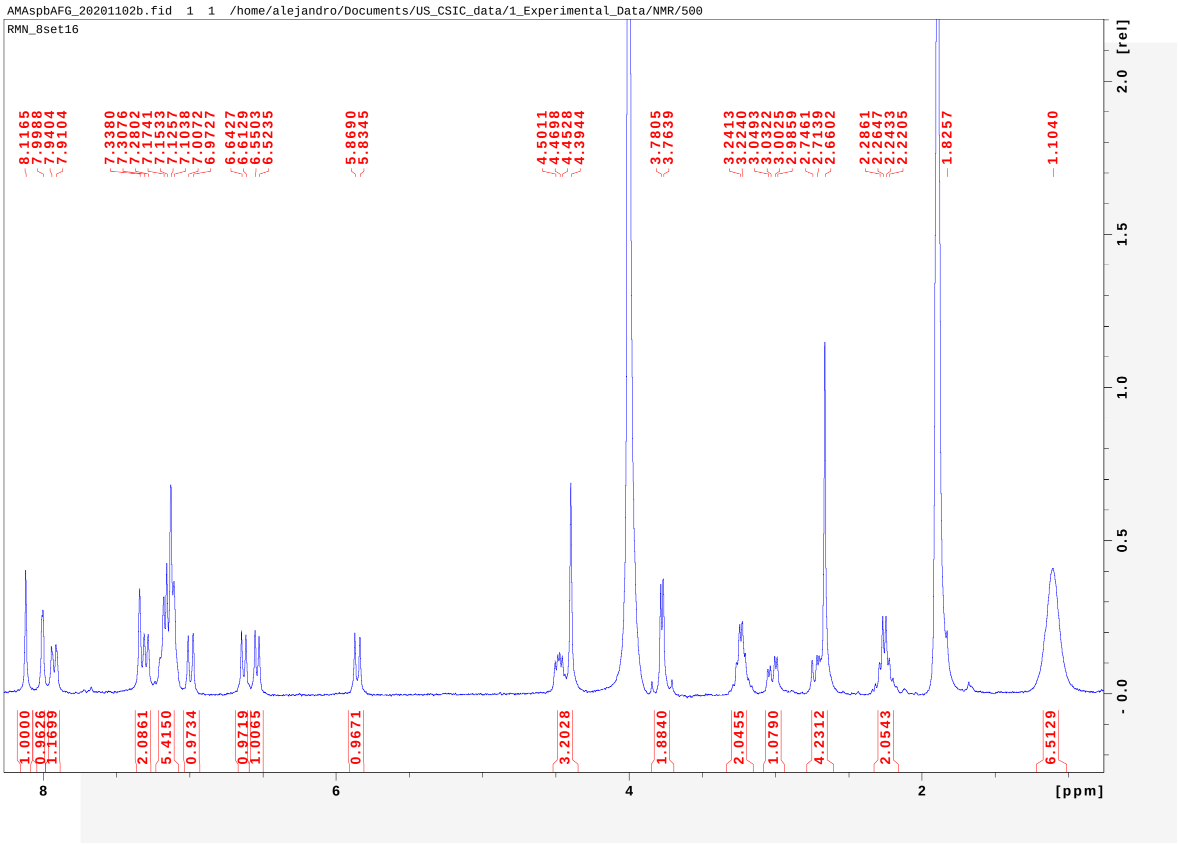

Figure S37: ^{1}H NMR (300 MHz, CD_3CN-D_2O 1:1, 293 K) of derivative **SP**.

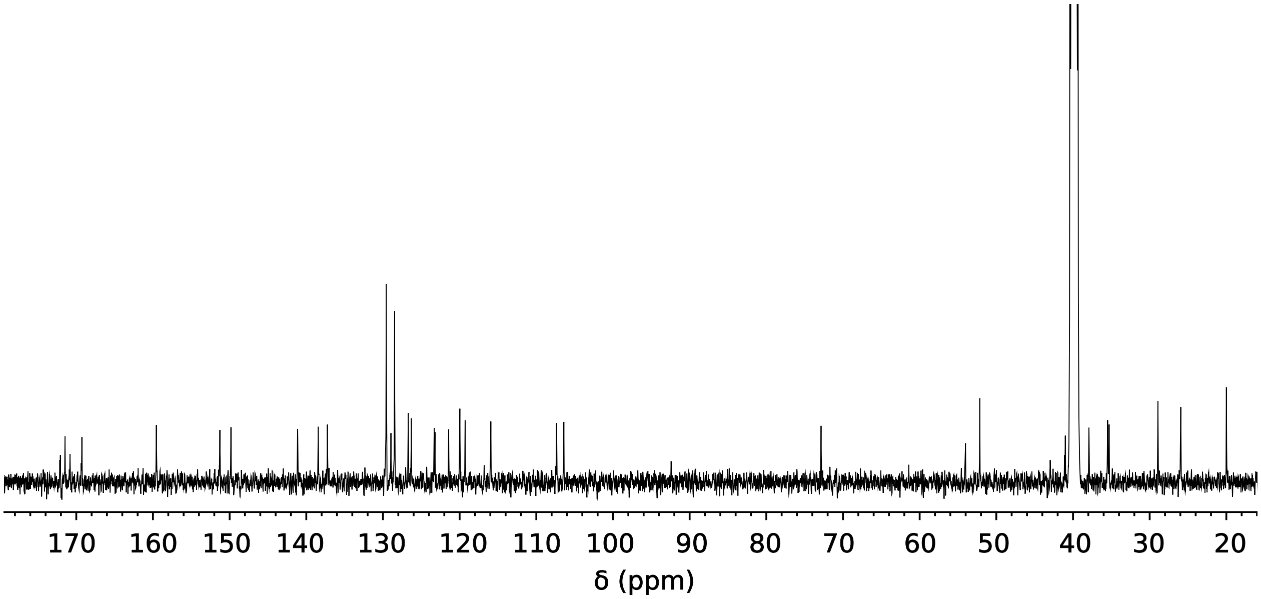

Figure S38: ^{13}C NMR (125.7 MHz, DMSO-d_6, 293 K) of derivative **SP**.

S3.3 SP (D)

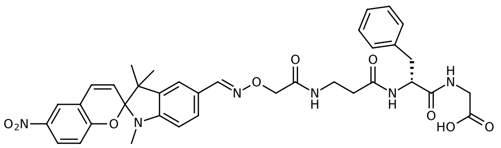

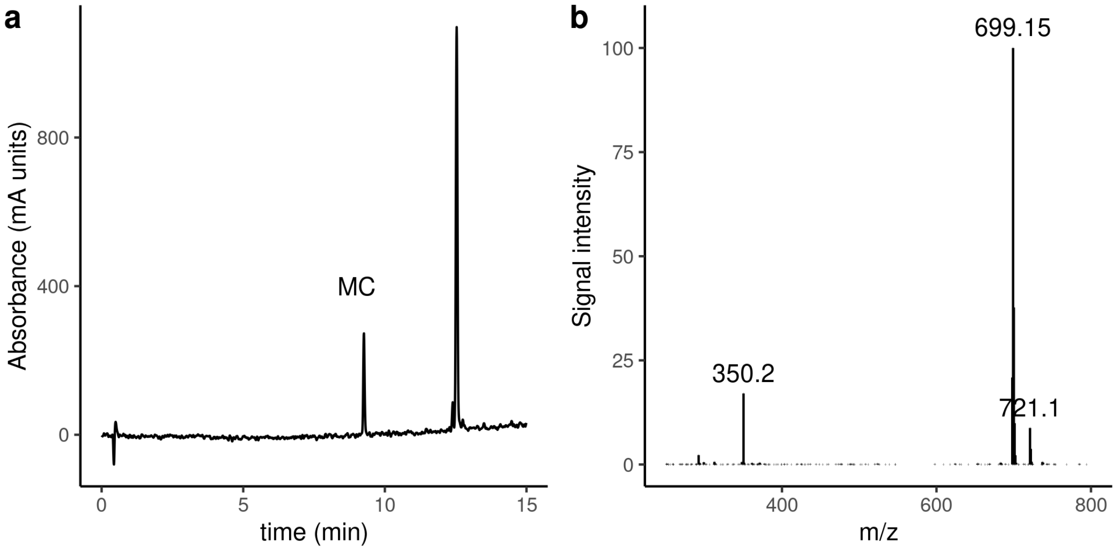

Figure S39: Chromatogram of derivative **SP (D)** (H_2O-0.1% TFA-acetonitrile-0.1% TFA 95:5 (0 min) to 5:95 (15 min)); b) Mass spectra of the more intense chromatographic band. Predicted mass for $C_{36}H_{39}N_6O_9^+$: 699.28 amu.

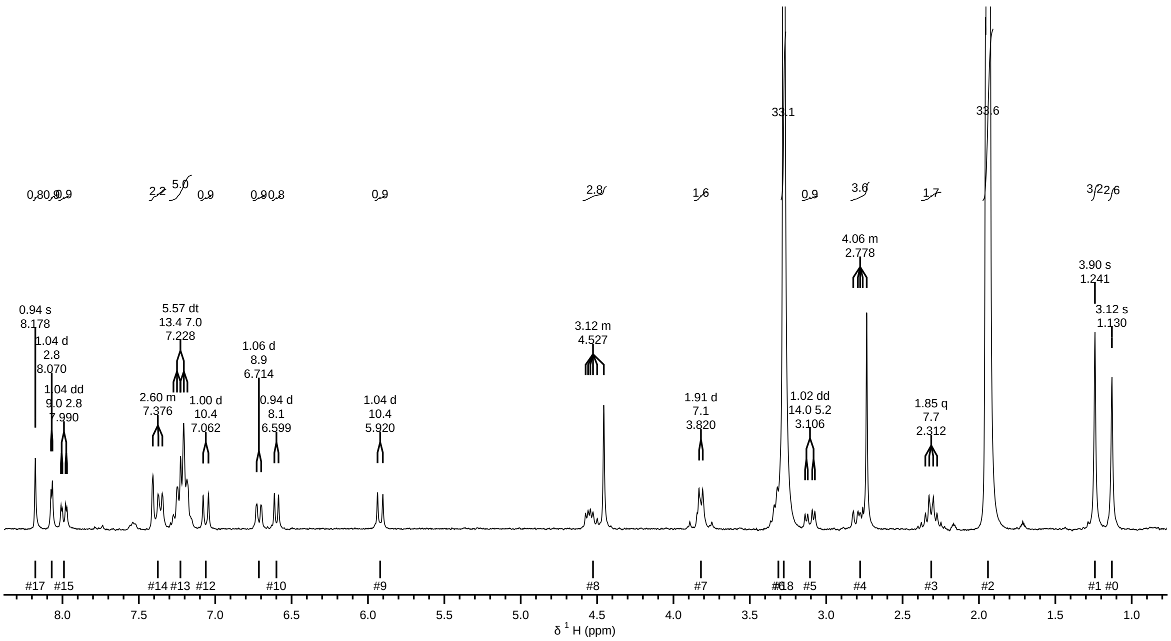

Figure S40: ^{1}H NMR (300 MHz, CD_3CN (few drops of D_2O) , 293 K) of derivative **SP (D)**

S3.4 βAAG

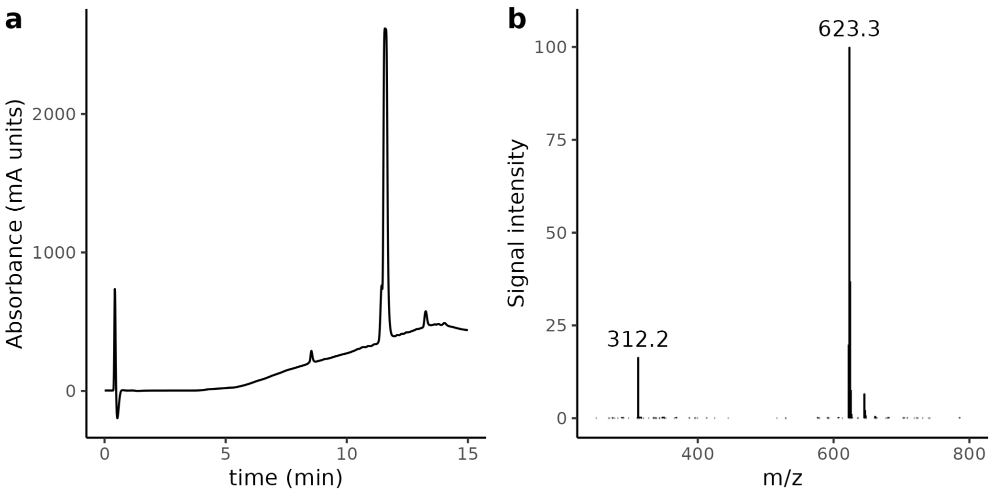

Figure S41: a) Chromatogram of peptide **bAFG** (H_2O-0.1% TFA-acetonitrile-0.1% TFA 95:5 (0 min) to 5:95 (15 min)); b) Mass spectra of the more intense band. Predicted mass for $C_{30}H_{35}N_6O_9^+$: 623.25 amu.

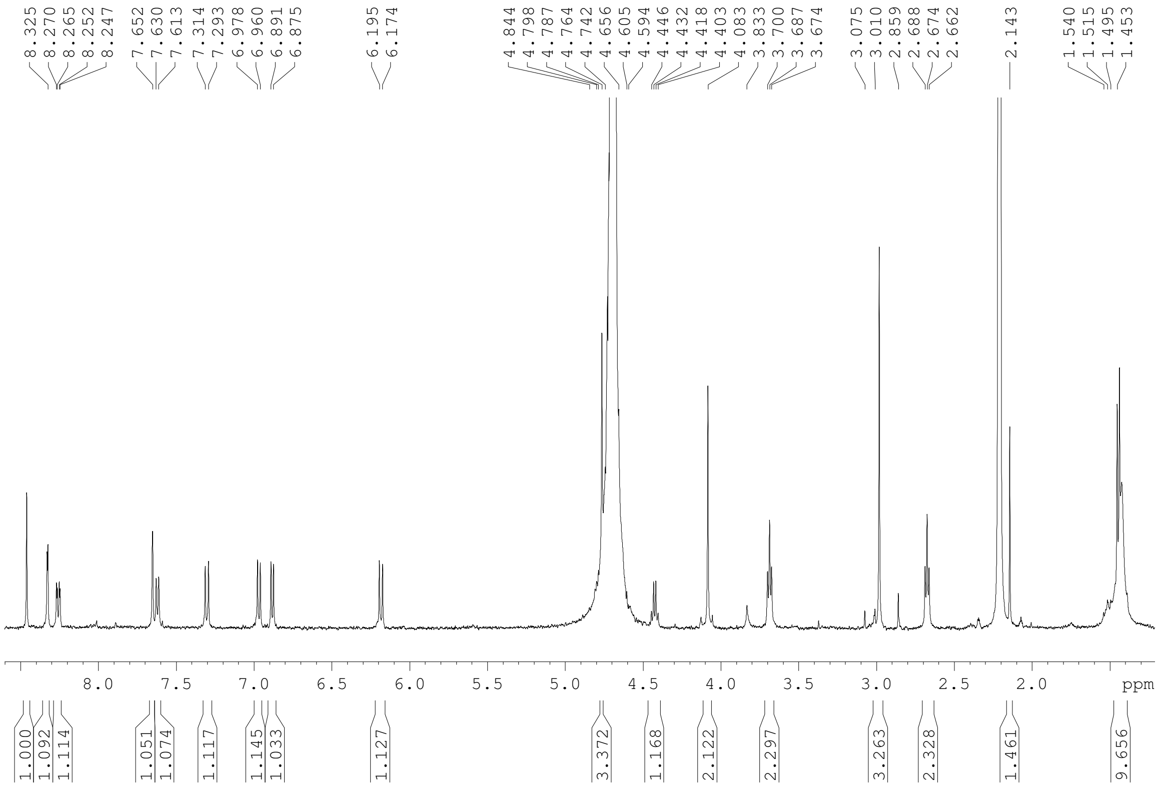

Figure S42: ^{1}H NMR (500 MHz, D_2O-CD_3CN 1:1, 298 K) of peptide β**AAG**

S3.5 βAVG

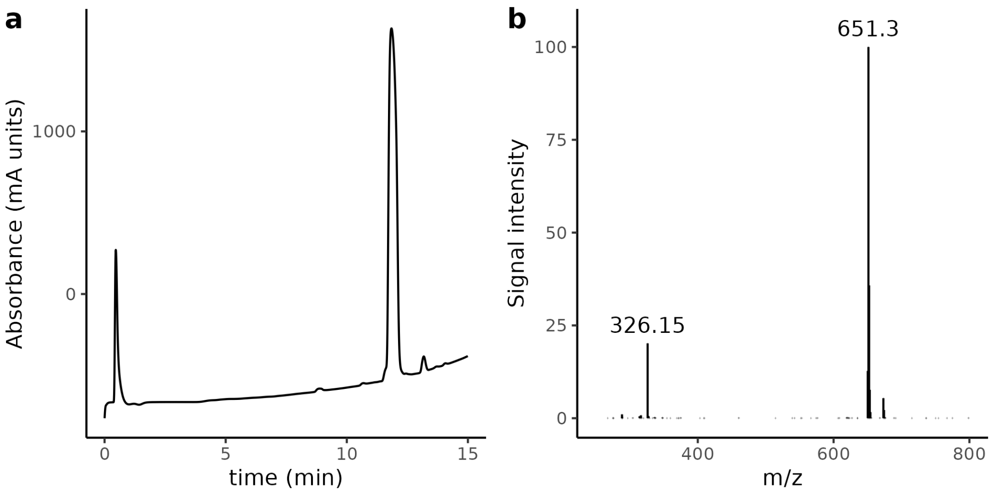

Figure S43: a) Chromatogram of peptide β**AVG** (H_2O-0.1% TFA-acetonitrile-0.1% TFA 95:5 (0 min) to 5:95 (15 min)); b) Mass spectra of the more intense band. Predicted mass for $C_{32}H_{39}N_6O_9^+$: 651.28 amu.

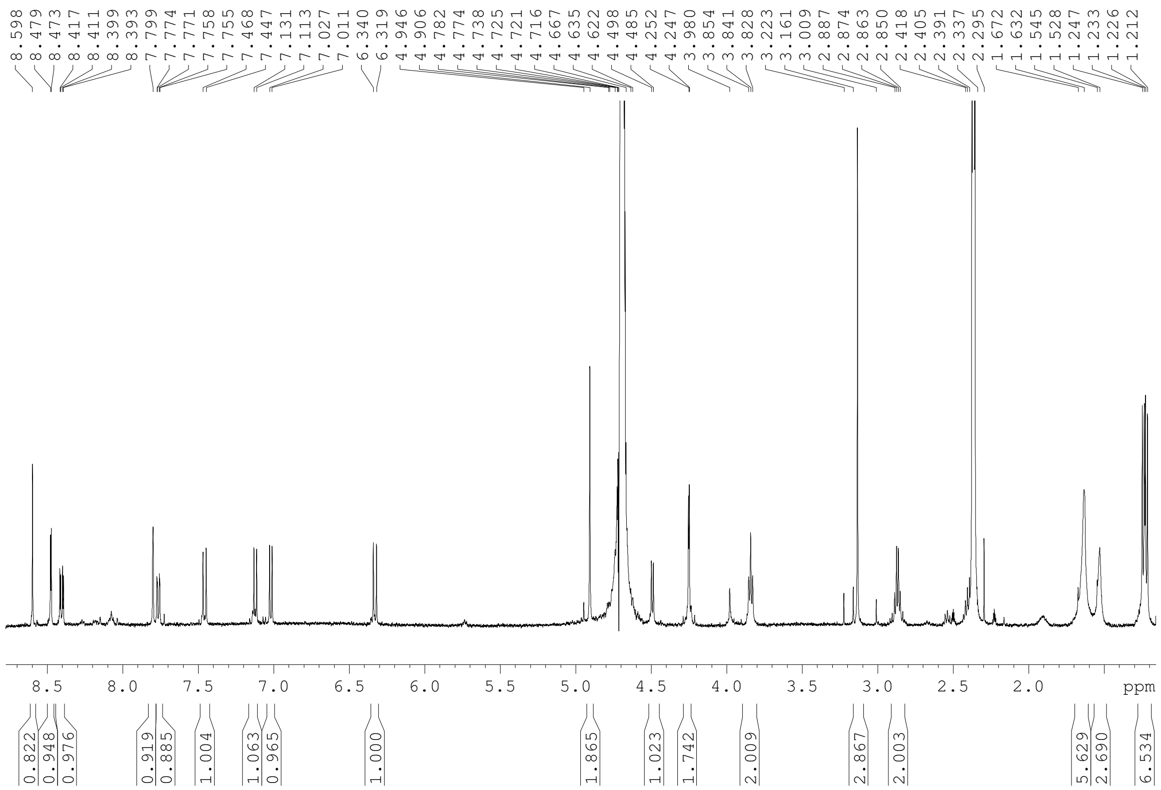

Figure S44: ^{1}H NMR (500 MHz, D_2O-CD_3CN 1:1, 298 K) of peptide β**AVG**

S3.6 βAGF

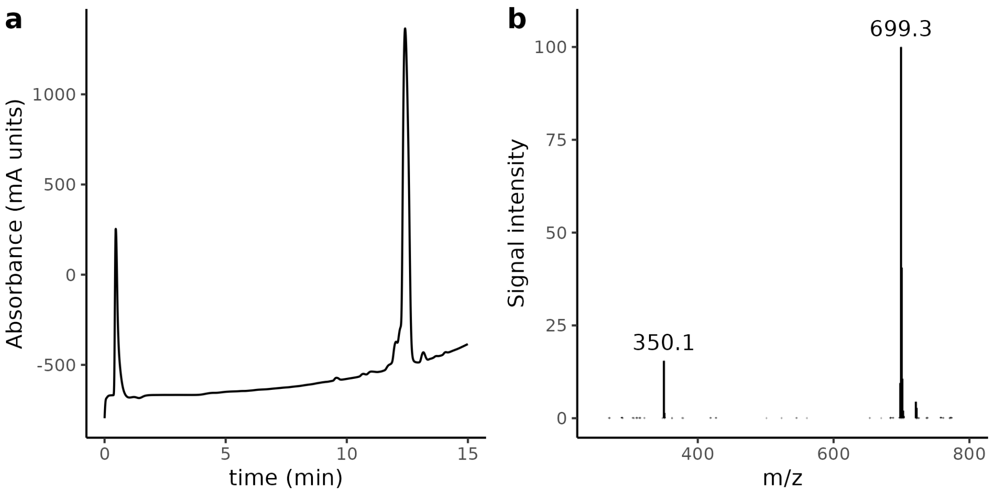

Figure S45: a) Chromatogram of peptide β**AGF** (H_2O-0.1% TFA-acetonitrile-0.1% TFA 95:5 (0 min) to 5:95 (15 min)); b) Mass spectra of the more intense band. Predicted mass for $C_{36}H_{39}N_6O_9^+$: 699.28 amu.

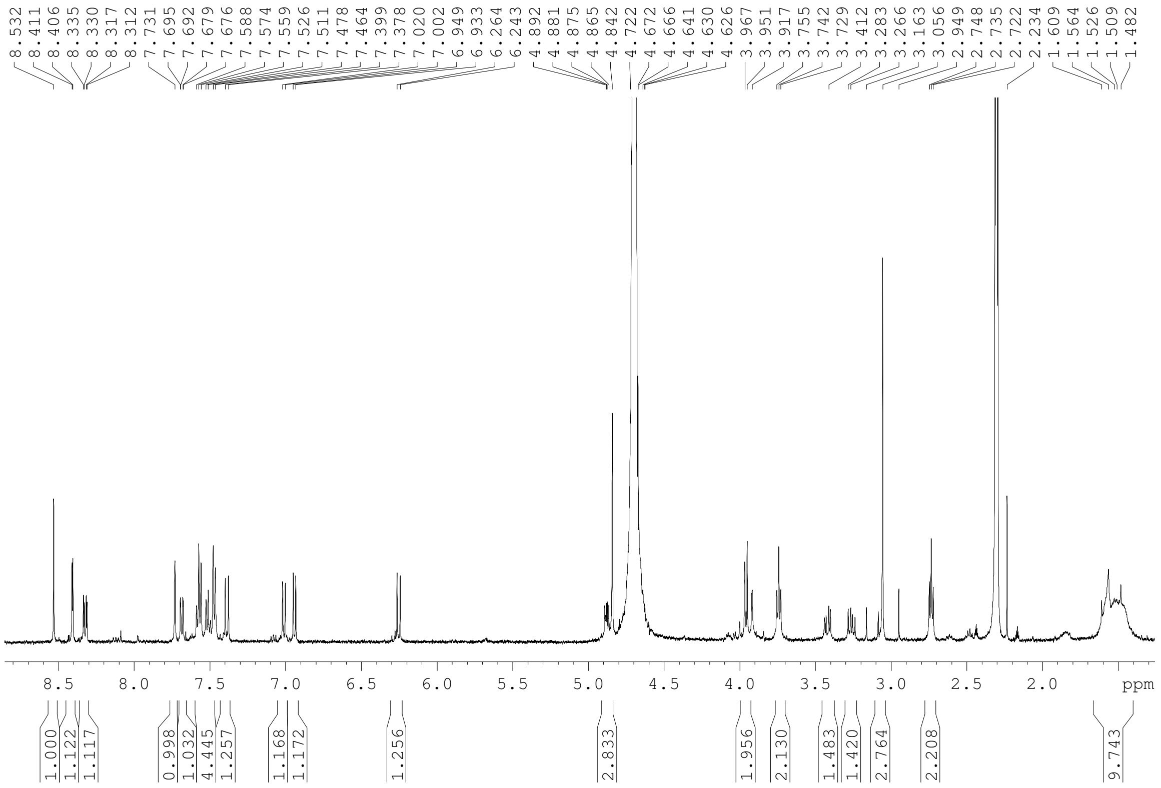

Figure S46: ^{1}H NMR (500 MHz, D_2O-CD_3CN 1:1, 298 K) of peptide β**AGF**

S3.7 GFG

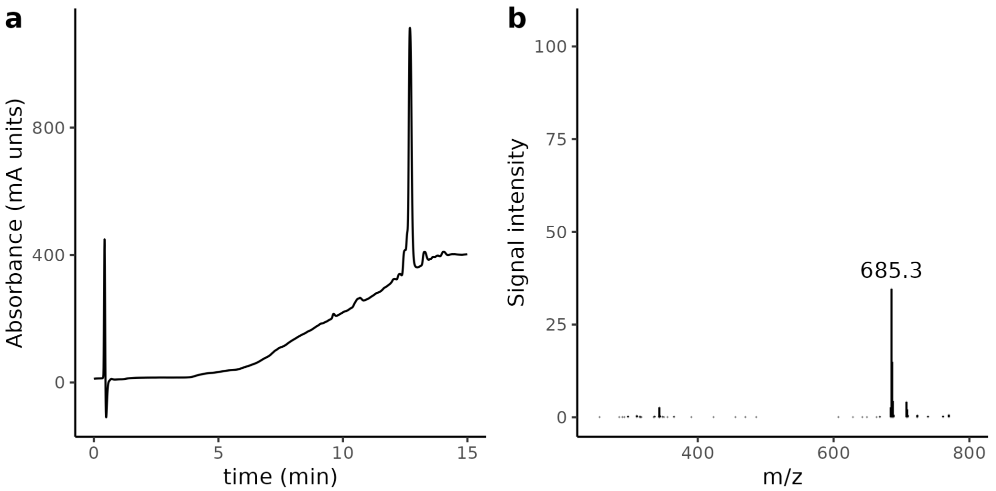

Figure S47: a) Chromatogram of peptide **spGFG** (H_2O-0.1% TFA-acetonitrile-0.1% TFA 95:5 (0 min) to 5:95 (15 min)); b) Mass spectra of the more intense band. Predicted mass for $C_{35}H_{37}N_6O_9^+$: 685.26.

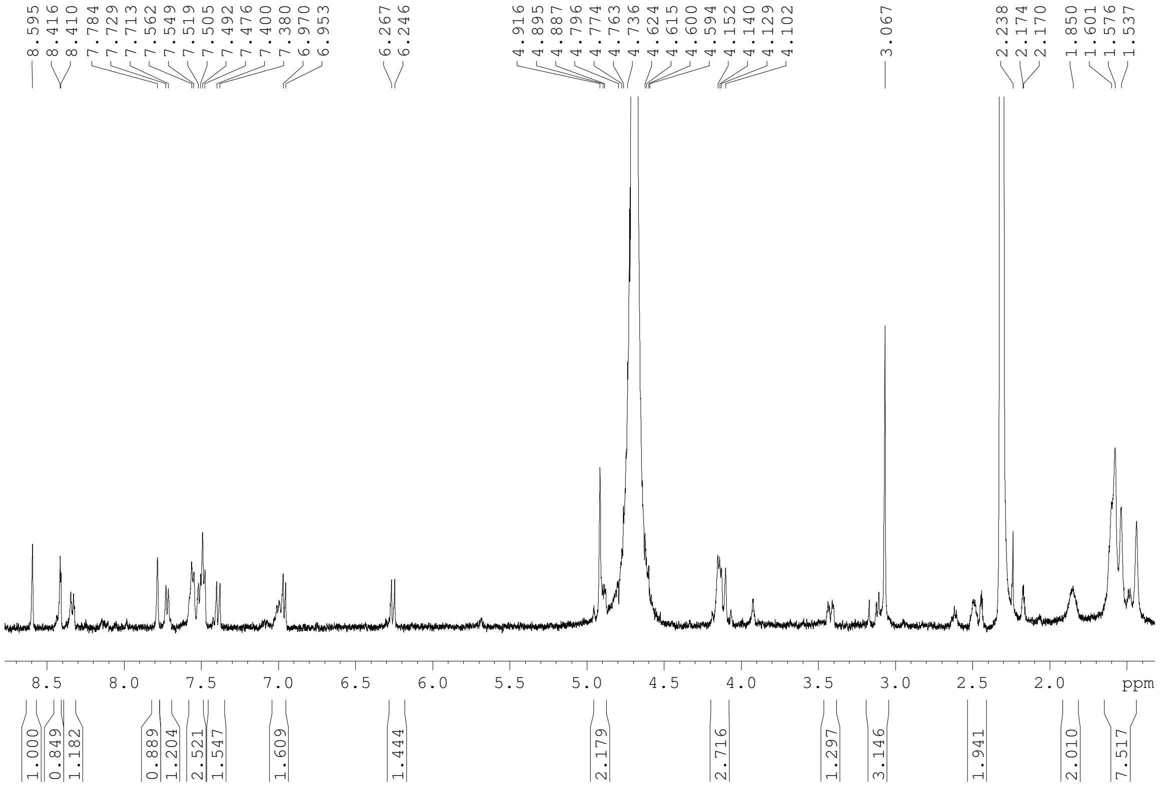

Figure S48: ^{1}H NMR (500 MHz, D_2O-CD_3CN 1:1, 298 K) of peptide **GFG**

S3.8 βAFE

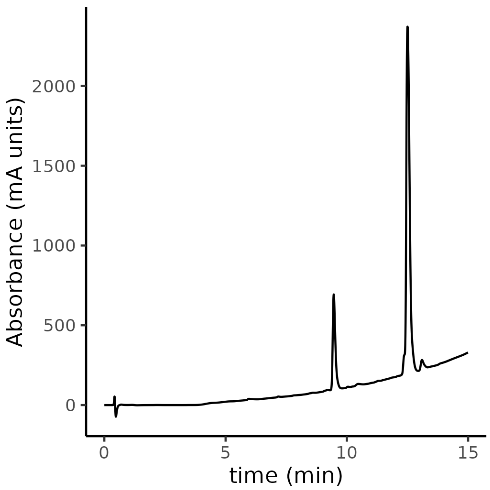

Figure S49: a) Chromatogram of peptide ***β*-AFE** (H_2O-0.1% TFA-acetonitrile-0.1% TFA 95:5 (0 min) to 5:95 (15 min))

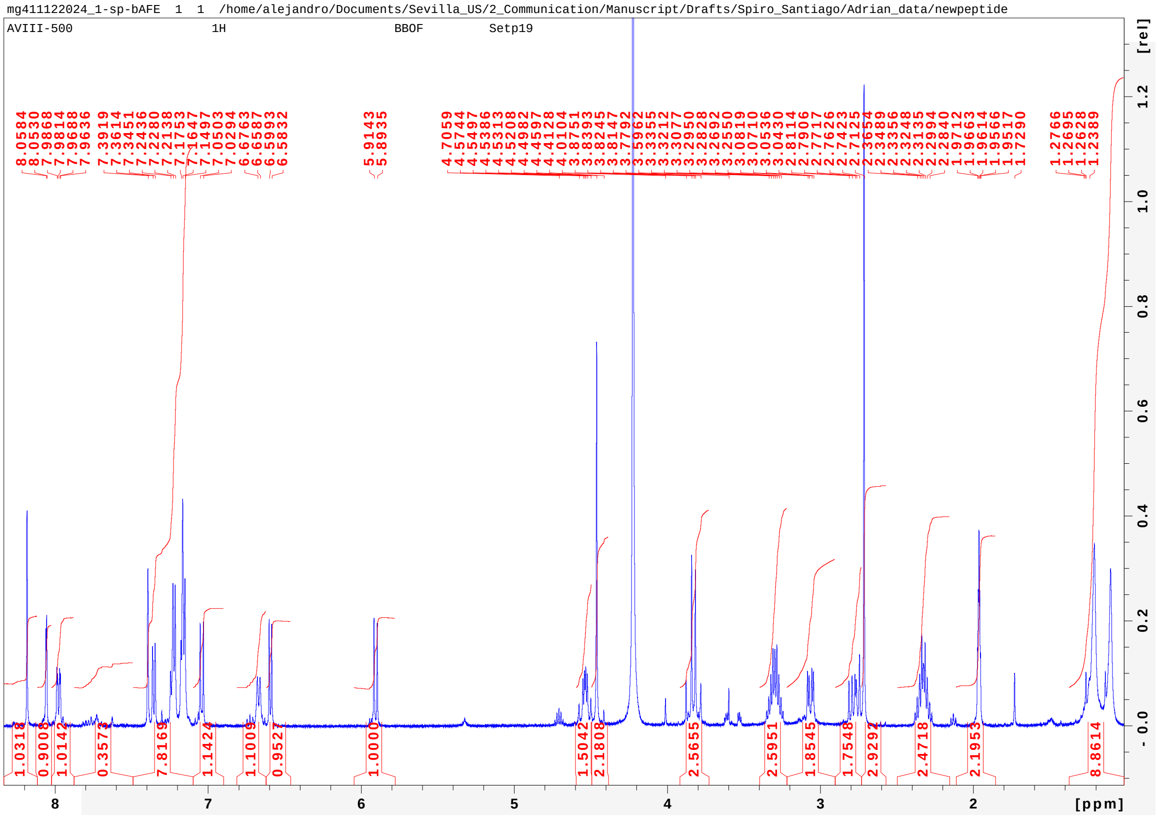

Figure S50: ^{1}H NMR (500 MHz, CD_3CN, 298 K) of peptide ***β*-AFE**

S4 References

(51) Laptev, A. V.; Lukin, A. Y.; Belikov, N. E.; Zvezdin, K. V.; Demina, O. V.; Barachevsky, V. A.; Varfolomeev, S. D.; Khodonov, A. A.; Shvets, V. I. *Russ. Chem. Bull.* **2014**, *63*, 20262035.

(52) El-Faham, A.; Albericio, F. *Chem. Rev.* **2011**, *111*, 65576602.

(53) Margolis, M., *Arduino cookbook*; O'Reilly: Sebastopol, Calif, 2012.

(54) Würthner, F.; Thalacker, C.; Diele, S.; Tschierske, C. *Chem. Eur. J.* **2001**, *7*, 22452253.

(55) Nakakoshi, M.; Nishioka, H.; Katayama, E. *J. Electron Microsc. (Tokyo)* **2011**, *60*, 401407.

(56) Schindelin, J.; Arganda-Carreras, I.; Frise, E.; Kaynig, V.; Longair, M.; Pietzsch, T.; Preibisch, S.; Rueden, C.; Saalfeld, S.; Schmid, B.; Tinevez, J.-Y.; White, D. J.; Hartenstein, V.; Eliceiri, K.; Tomancak, P.; Cardona, A. *Nat. Methods* **2012**, *9*, 676682.

(57) Schneider, C. A.; Rasband, W. S.; Eliceiri, K. W. *Nat. Methods* **2012**, *9*, 671675.

(58) Neas, D.; Klapetek, P. *Cent. Eur. J. Phys.* **2012**, *10*, 181–188.

(59) Watzky, M. A.; Finke, R. G. *J. Am. Chem. Soc.* **1997**, *119*, 1038210400.

(60) R Core Team R: A Language and Environment for Statistical Computing; R Foundation for Statistical Computing, Vienna, Austria, 2020.

(61) Wickham, H., *ggplot2: Elegant Graphics for Data Analysis*; Springer-Verlag New York: 2016.

(62) Baty, F.; Ritz, C.; Charles, S.; Brutsche, M.; Flandrois, J.-P.; Delignette-Muller, M.-L. *J. Stat. Softw.* **2015**, *66*, 121.

(63) Elzhov, T. V.; Mullen, K. M.; Spiess, A.-N.; Bolker, B. minpack.lm: R Interface to the Levenberg-Marquardt Nonlinear Least-Squares Algorithm Found in MINPACK, Plus Support for Bounds, R package version 1.2-1, 2016.
